# Poles Apart: Comparing Trends of Alien Hymenoptera in New Zealand with Europe (DAISIE)

**DOI:** 10.1371/journal.pone.0132264

**Published:** 2015-07-06

**Authors:** Darren Ward, Emma Edney-Browne

**Affiliations:** 1 New Zealand Arthropod Collection, Landcare Research, Private Bag 92170, Auckland, New Zealand; 2 School of Biological Sciences, University of Auckland, Private Bag 92019, Auckland, New Zealand; Institut National de la Recherche Agronomique (INRA), FRANCE

## Abstract

Developing generalisations of invasive species is an important part of invasion biology. However, trends and generalisations from one part of the world may not necessarily hold elsewhere. We present the first inventory and analysis of all Hymenoptera alien to New Zealand, and compare patterns from New Zealand with those previously published from Europe (DAISIE). Between the two regions there was broad correlation between families with the highest number of alien species (Braconidae, Encyrtidae, Pteromalidae, Eulophidae, Formicidae, Aphelinidae). However, major differences also existed. The number of species alien to New Zealand is higher than for Europe (334 vs 286), and major differences include: i) the much lower proportion of intentionally released species in New Zealand (21% vs 63% in Europe); and ii) the greater proportion of unintentionally introduced parasitoids in New Zealand (71.2% vs 22.6%). The disharmonic ‘island’ nature of New Zealand is shown, as a high proportion of families (36%) have no native representatives, and alien species also represent >10% of the native fauna for many other families. A much larger proportion of alien species are found in urban areas in New Zealand (60%) compared to Europe (~30%), and higher numbers of alien species were present earlier in New Zealand (especially <1950). Differences in the origins of alien species were also apparent. Unlike Europe, the New Zealand data reveals a change in the origins of alien species over time, with an increasing dominance of alien species from Australasia (a regional neighbour) during the past 25 years. We recommend that further effort be made towards the formation, and analysis, of regional inventories of alien species. This will allow a wider range of taxa and regions to be examined for generalisations, and help assess and prioritise the risk posed by certain taxa towards the economy or environment.

## Introduction

Intensification of human transportation and commerce around the world has led to the movement of many species outside of their native range [1,2]. As a result, biological invasions are now a global phenomenon, and are widely recognised as a significant component of global change, affecting agro-forestry industries, natural ecosystems, and social activities [1–3]. Developing generalisations of invasive species is therefore a key concept for invasion biology theory. However, biological invasions are driven by a complex interaction of biogeography [4], socio-economic issues (both current and historical) [5], global trade dynamics [6], and the distribution and densities of human populations [7]. As a consequence, trends and generalisations from one part of the world may not necessarily hold elsewhere.

Despite forming a large part of the alien (non-indigenous) fauna worldwide, invertebrates have received disproportionality less attention compared with the impacts of plants and vertebrates, especially for impacts associated with native biodiversity [8,9]. Much of the historic work concerning alien invertebrates has focused on case studies or syntheses of important economic or sanitary pests [10,11]. However, in recent years there has been a growing awareness of the wider environmental impacts of alien invertebrates [8,9,12–14], and the need for a broader scope and taxonomic coverage in order to identify and analyse large-scale trends [15,16].

Hymenoptera is one of the four megadiverse insect orders in the world, with the number of described species exceeding 100 000 [17]. Hymenoptera exhibit a very diverse range of groups (social wasps, bees, ants, sawflies, and parasitic wasps) and these play extremely important ecological and economic roles. For example, bees provide vital pollination services to natural and managed systems, and parasitic wasps are commonly used to suppress populations of pest insects in agriculture and forestry sectors [10,17–19]. However, some phytophagous Hymenoptera can be major pests to plantation forests and in horticulture [17,20,21]. Invasive ants can have huge economic costs and disrupt populations and communities of native species [22], while stings from social wasps and bees impact on human health and recreational activities [12].

For Hymenoptera, as with other invertebrate groups, information is often lacking on invasion history, so that analyses on pathways and establishment patterns can be difficult [4,15,23]. However, the development of the DAISIE project (Delivering Alien Invasive Species Inventories for Europe) has shown the importance of large-scale taxonomic inventories of alien species. Together with key information, such as introduction dates and invaded localities, inventories allow trends of alien species to be examined, and contribute to determining the relative importance of the different taxa as invaders, or which ecosystems or habitats are most at risk [16].

New Zealand is well known for the negative impacts of alien species in natural environments, especially mammalian pests [24]. For invertebrates in natural environments, much work has focused on *Vespula* wasps [25,26], but recent work also highlights invasive ants [27,28] and *Polistes* wasps as threats [29,30]. These examples, however, are all of predatory species, and only represent a small fraction of alien Hymenoptera in New Zealand. Very little is known about the large number of other alien species (especially phytophagous and parasitoid species) and whether they are having negative impacts in the natural environment (or could in the future).

The main aim of this paper is to present an inventory of all alien Hymenoptera in New Zealand, and also to analyse spatial and temporal trends across a large number of species. An additional aim is to compare the alien species of Hymenoptera in New Zealand with that of Europe (from the DAISIE project [31]) to see if there are consistent generalisations or trends across different regions.

## Materials and Methods

### Study Area and Fauna

New Zealand comprises two large islands (North Island, and South Island) that span latitudes of 34–47°S, which have a cool to warm temperate climate with a strong maritime and orographical influence [32]. The New Zealand Hymenoptera fauna is relatively poorly known [33,34] but is considered unusual particularly for its near absence of sawflies, depauperate Aculeate fauna, and a very high diversity of Diapriidae and Mymaridae [35–39]. Species-level endemism is high (~90%) but there is an absence of many higher taxonomic levels [40].

### Data Collection

We compiled a list of all Hymenoptera that are alien to New Zealand (see Appendix 1), chiefly based on a 2010 checklist [40] but supplemented with other literature and examination of specimens in the New Zealand Arthropod Collection (NZAC). Species were categorised as either ‘intentionally released’ or ‘unintentional introductions’ (via containment and stowaway pathways [41]). Information on intentionally released species (e.g. pollinators, or biological control agents) was obtained from the ‘Biological Control Agents introduced to New Zealand’ website, dedicated to summarising information on intentional releases of alien species [42]. We used primary literature searches and examined specimens to obtain species-level information on a number of variables, and used Chi square tests and contingency tables to analyse the following four datasets.

1. **Distribution and Invaded habitats**. We determined the approximate distributions of alien species in New Zealand (North and South Islands) by recording localities from specimen labels in the NZAC. Species were categorised into a geographic region [43] (maximum number of regions = 28). We also utilised georeferenced locality information to examine landcover and habitat associations of alien species. We used the ‘Land Cover Database v1, 1997’ [44], and broadly categorised land cover as: i) built-up areas; ii) primary productive land (agriculture, horticulture, or forestry); iii) native habitats; iv) coastal or river habitats; and v) alpine habitats. Although the NZAC has voucher specimens of first releases for intentionally released species, it does not often have subsequent records of spread, and distribution information is thus incomplete. Therefore, we did not analyse the distribution of intentionally released species. We determined the distributions for 79.5% of unintentionally introduced species (210/264).
2. **Temporal trends**. We used the earliest date a species was first in New Zealand as the date of introduction. Dates for intentionally released species are very precise. Dates for unintentional introductions are based on the best available information, as species may have been introduced several years before they were reported. Several species were first recorded from more than one location, and these were excluded from analysis. We determined a date of introduction for 93.4% (312/334) of alien species introduced to New Zealand.
3. **Origins**. Information on the native origin of a species was obtained through searches of primary literature and Hymenoptera databases (Taxpad [45] and the Universal Chalcidoidea Database [46]). It was not usually possible to determine a specific country of origin, so we used biogeographic regions (African, Australasia, Holarctic, Neotropical, Oriental, and Cosmopolitan). We determined a region of origin for 97.6% (326/334) alien species introduced to New Zealand.
4. **Border interceptions**. Information on Hymenoptera intercepted at the New Zealand border was obtained from the Ministry of Primary Industries (based on >3900 records, from 1955–1982). We summarised information at the family-level, as identifications at this taxonomic level were the most stable over time, and genus-level identifications were not often made, especially for Parasitica.

## Results

### Taxonomy of alien species

**Fig 1:**
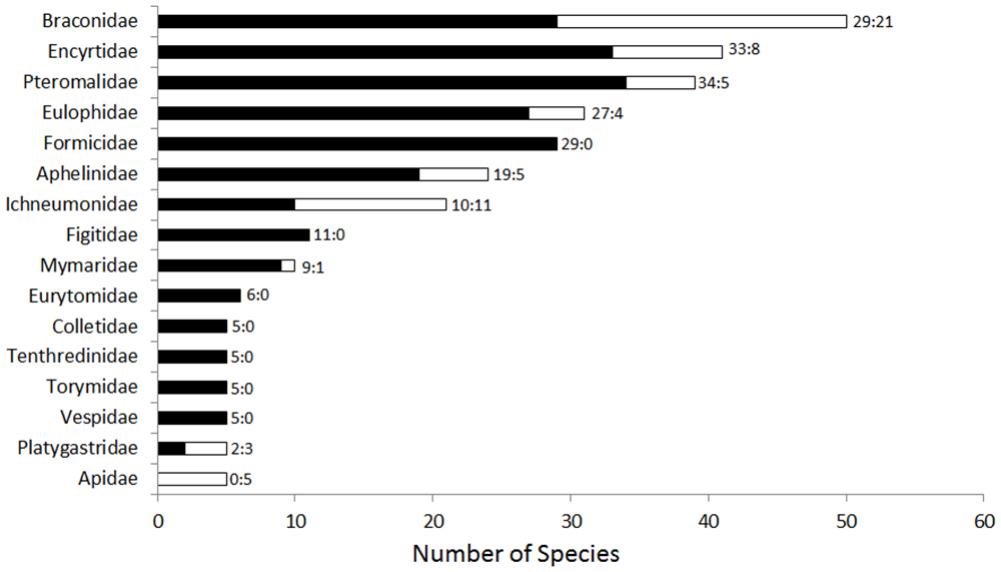
Taxonomic overview of the alien Hymenoptera in New Zealand. Families are presented in a decreasing order based on the number of alien species. Black = unintentional introductions, white = intentional releases. Numbers at end of bars are number of species for unintentional introductions vs intentional releases. Families with less than five species are excluded from the figure (4 species: Bethylidae, Megaspilidae; 3 species: Chalcididae, Megachilidae, Signiphoridae; 2 species: Agaonidae, Crabronidae, Cynipidae, Diapriidae, Halictidae, Scelionidae, Trichogrammitidae; 1 species: Dryinidae, Eupelmidae, Ibaliidae, Mutillidae, Pergidae, Pompilidae, Proctotrupidae, Scolebythidae, Scoliidae, Siricidae, Sphecidae).

We determined that 334 species of Hymenoptera, from 39 different families, are alien to New Zealand, (Fig 1; S1 and S2 Tables). Most alien Hymenoptera in New Zealand belong to the Parasitica (266 spp., 22 families, 79.6% of the species), while Aculeata (61 spp., 14 families, 18.3%) and Symphyta (7 spp., 3 families, 2.1%) are less represented.

Intentional releases represent an important proportion of these alien species (n = 70, 21%; Fig 1), mostly due to the high number of parasitoids released as biological control agents (n = 61) versus releases of pollinators or phytophagous species (n = 9). Among the 264 species unintentionally introduced into New Zealand, 38 are pollinators or phytophagous species (14.4% of total); a further 38 species (14.4%), are predators, while the majority are parasitoids or hyper-parasitoids (71.2%).

**Fig 2:**
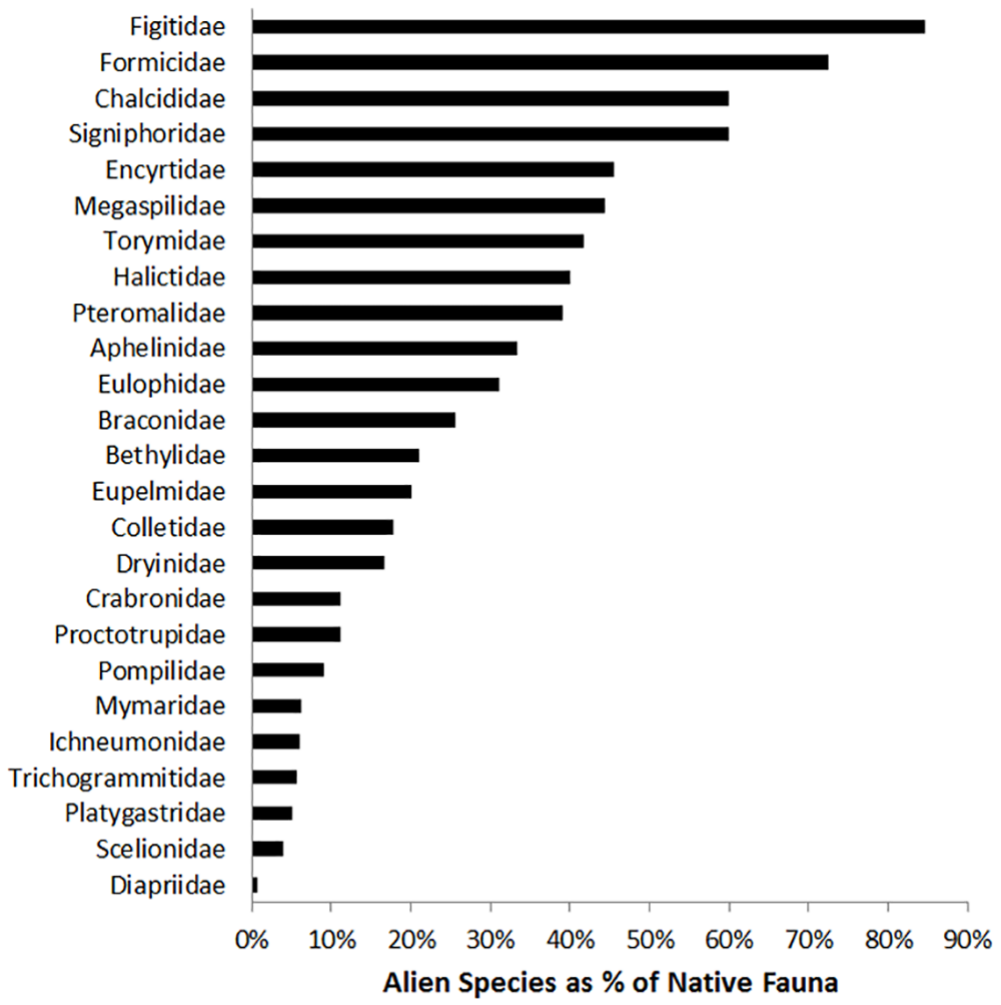
Alien Hymenoptera as a proportion of the New Zealand native fauna. Fourteen families are also alien in New Zealand without native representatives (Agaonidae, Apidae, Cynipidae, Eurytomidae, Ibaliidae Megachilidae, Mutillidae, Pergidae, Scolebythidae, Scoliidae, Siricidae, Sphecidae, Tenthredinidae, and Vespidae).

The top 7 families represent 70% of the alien Hymenoptera, (Braconidae, Encyrtidae, Pteromalidae, Eulophidae, Formicidae, Aphelinidae, Ichneumonidae; Fig 1). Fourteen families (36% of families) are alien in New Zealand without native representatives (Fig 2). Several of these are intentional releases (Apidae, Cynipidae in part, Ibaliidae, and Megachilidae in part,), but most are not (Agaonidae, Cynipidae in part, Eurytomidae, Megachilidae in part, Mutillidae, Pergidae, Scolebythidae, Scoliidae, Siricidae, Sphecidae, Tenthredinidae, and Vespidae). Another 18 families also have the alien species as >10% of the native fauna (Fig 2).

### Distribution and Invaded habitats

**Fig 3:**
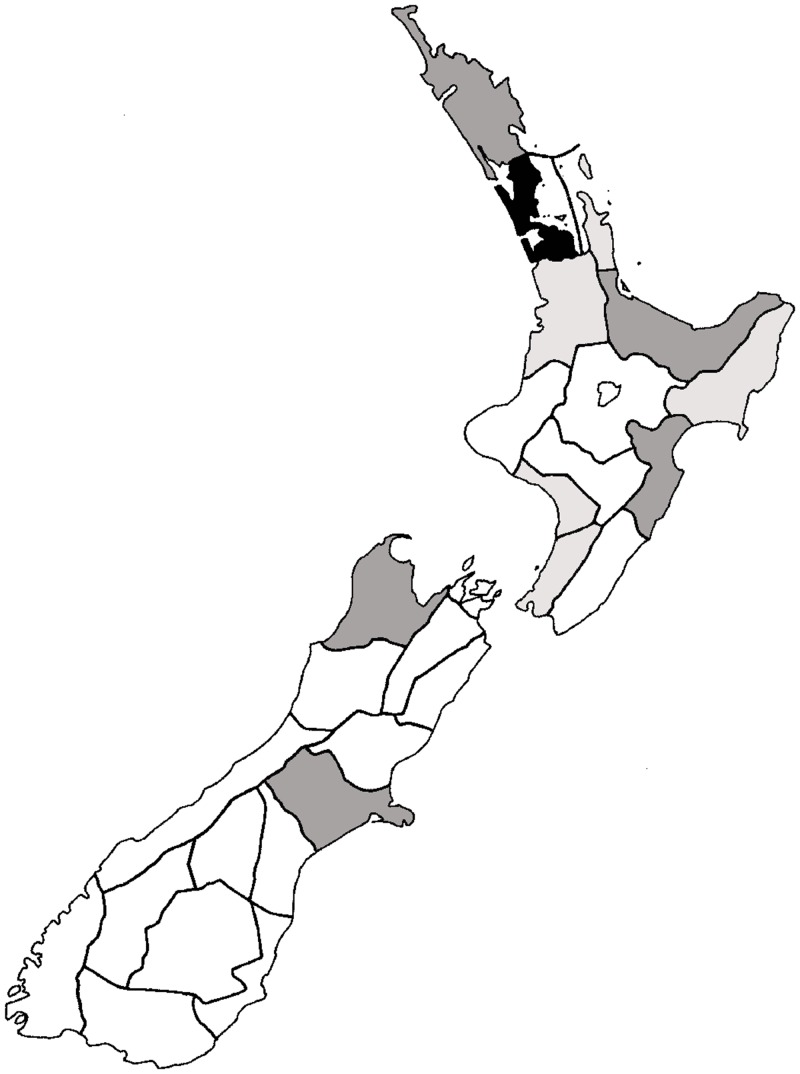
Distribution of alien Hymenoptera throughout New Zealand regions. White = 0–25; light grey = 26–5; dark grey = 51–75; and black = 76–100+ species. Further details of the regions can be found in Crosby et al. (1998).

Alien Hymenoptera species are not evenly distributed throughout New Zealand (Chi square = 888.202, d.f. = 27, p < 0.001; Fig 3). The Auckland region has the highest number of unintentional alien species (162 species), which is double that of the next region (Nelson). The top 5 regions have 47.6% of alien species and 11 regions contributed 75% of alien species.

A large proportion of records (60.8%) of unintentional alien Hymenoptera in New Zealand come from built-up areas (including residential areas, and urban parkland/open spaces). Primary productive land (agriculture, horticulture, forestry) is next with 19.2% of records, while native habitats (including forest and scrub) make up 17% of records.

### Temporal trends

**Fig 4:**
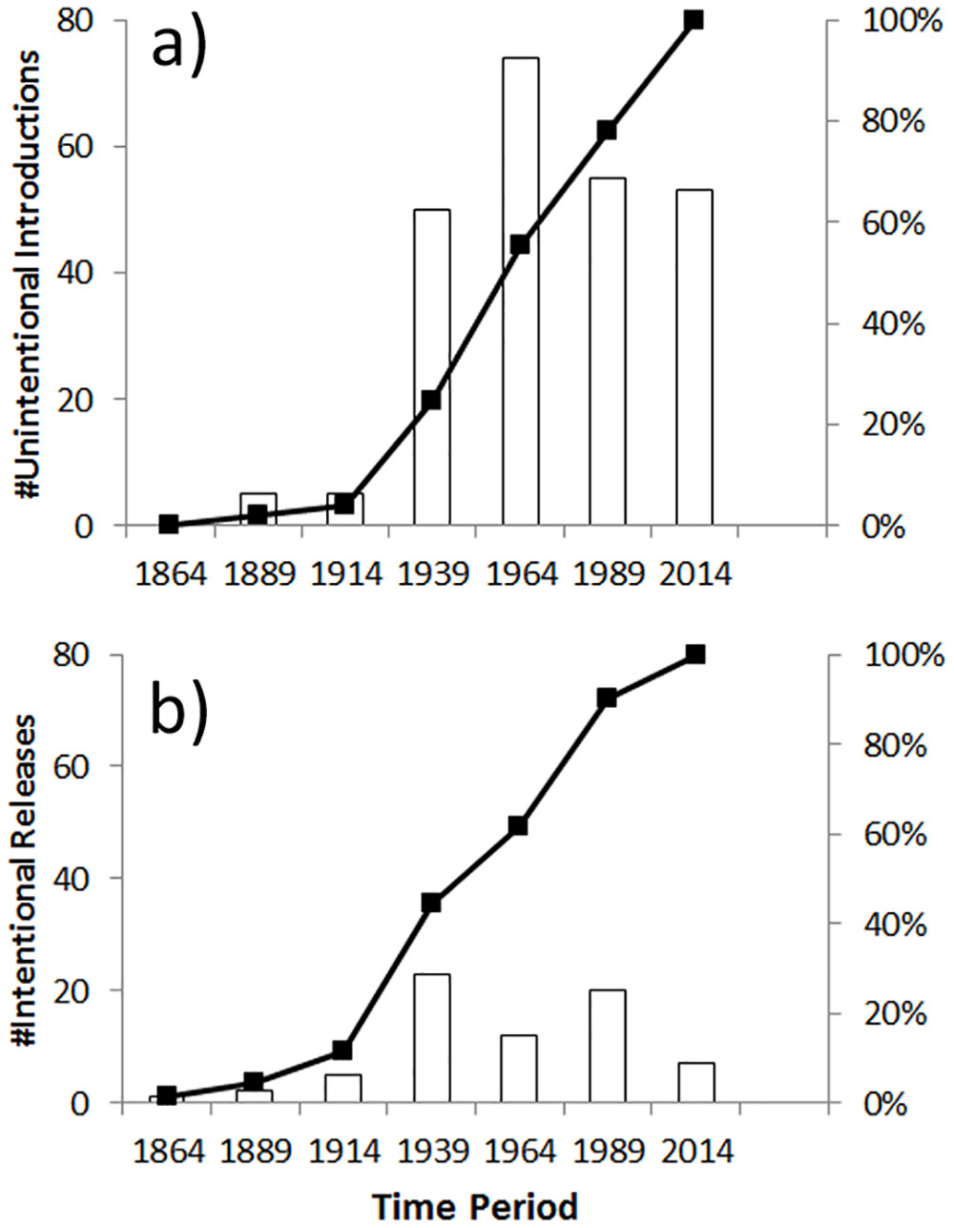
Timelines of introductions of alien Hymenoptera established in New Zealand. a) Unintentional introductions. b) Intentionally released species. Bars represent the number of species; line represents percent accumulation of species. Dates are the ‘end point’ of the period, date range is 1840–2014.

There is an exponential increase in the cumulative number of alien Hymenoptera during the last ~170 years, with a rapid rise recorded after 1914 (Fig 4a). Patterns for intentionally released species have two peaks in the number of species (1915–1939 and 1965–1989) and this is significantly different from unintentionally introduced species Chi square = 23.912, d.f. = 5, p < 0.001; Fig 4b). These peaks match the main periods of ‘biological control programs’ in New Zealand horticulture (Charles 1998).

### Origins

**Fig 5:**
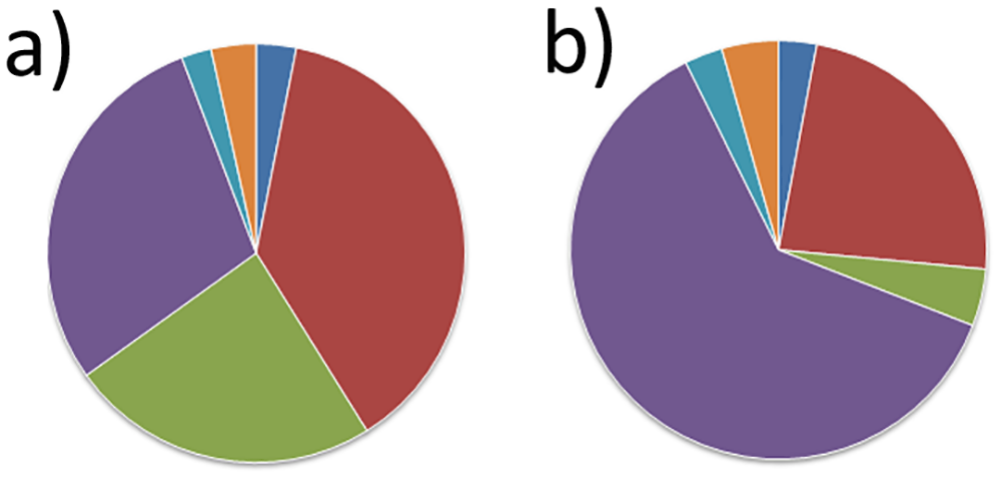
Origin of alien Hymenoptera established in New Zealand. a) Unintentional introductions. b) Intentionally released species. Dark blue = Africa; red = Australasia; green = Cosmopolitan; purple = Holarctic; light blue = Neotropical; and orange = Oriental.

Australasia (and principally Australia) provided the greatest part of unintentional alien Hymenoptera (98 species, 38.0%, Chi square = 325.518, d.f. = 5, p < 0.001), followed closely by the Holarctic region (75 species, 29.1%) and species considered cosmopolitan (62 species, 24.0%) (Fig 5a). The pattern for intentional releases is significantly different (Chi square = 40.122, d.f. = 5, p < 0.001; Fig 5b), with more species originating from the Holarctic region (42 species, 62%) and then Australasia (16 species, 24%).

**Fig 6:**
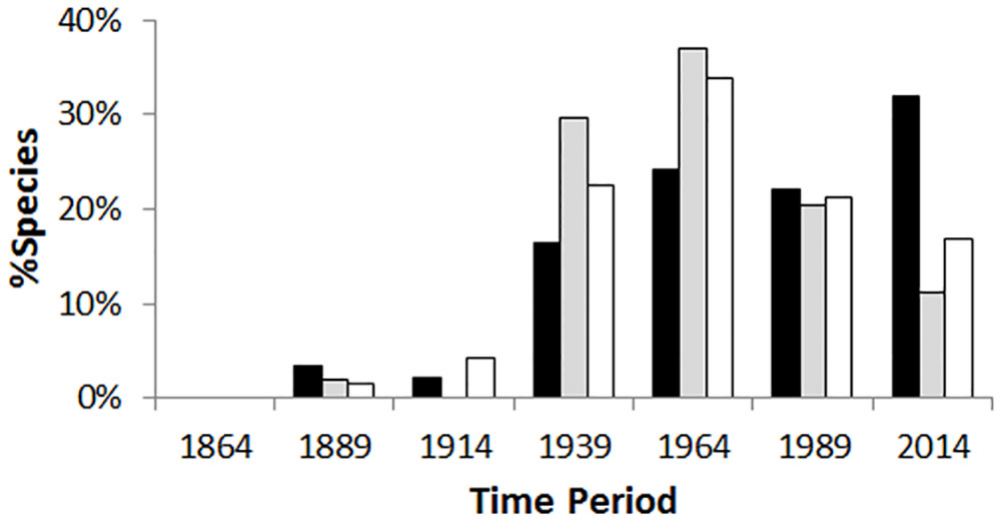
Changes in the origins of unintentionally introduced alien Hymenoptera through time. Black = Australasia; Grey = Cosmopolitan; White = Holarctic. Species intentionally released for biological control or pollination are excluded.

We also detected a significant change in the origins of alien species over time (Chi-square = 12.690; df = 6, p = 0.048). There is an increasing dominance of alien species from Australasia during the past 25 years (Fig 6). This represents the combined effect of not only an increased numbers of alien species from Australasia but also of a decline in the number of alien species from the Holarctic region and of cosmopolitan species.

### Border Interceptions

**Fig 7:**
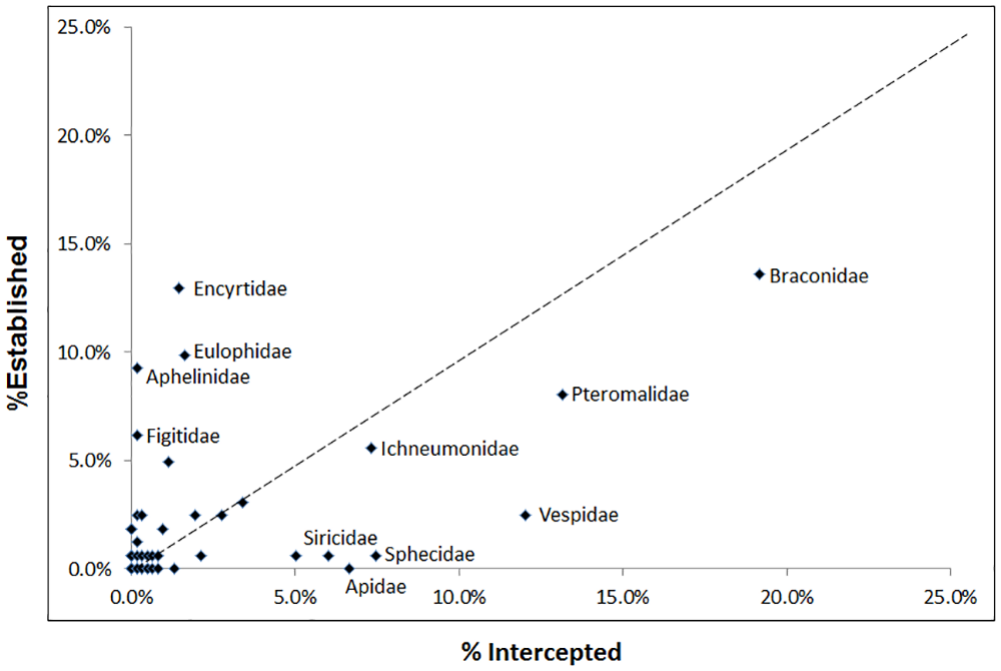
Family-level proportions of border interception records versus proportions of alien Hymenoptera establishment. Dotted line represents a 1:1 ratio in the proportion of border interceptions and the proportion of established species. Species intentionally released for biological control or pollination are excluded. Ants excluded to show the clarity of other families (ant co-ordinates are X = 84%, Y = 9%). Data obtained from Ministry of Primary Industries for the period 1955–1982.

Several families make up a high proportion of border interception records but are not well represented in established records (fall below the line; e.g. Apidae, Braconidae, Pteromalidae, Sphecidae, Siricidae, and Vespidae; Fig 7). Ants (Formicidae) also fall into this category; they make up a very large proportion of border interception records (~84%, averaged 1955–1982), but only represent 9% of established alien species. Conversely, a number of families have low proportions of border interception records yet are well represented in established species, for example, Aphelinidae, Encyrtidae, Eulophidae, and Figitidae (fall above the line; Fig 7).

### A comparison of New Zealand and Europe

**Fig 8:**
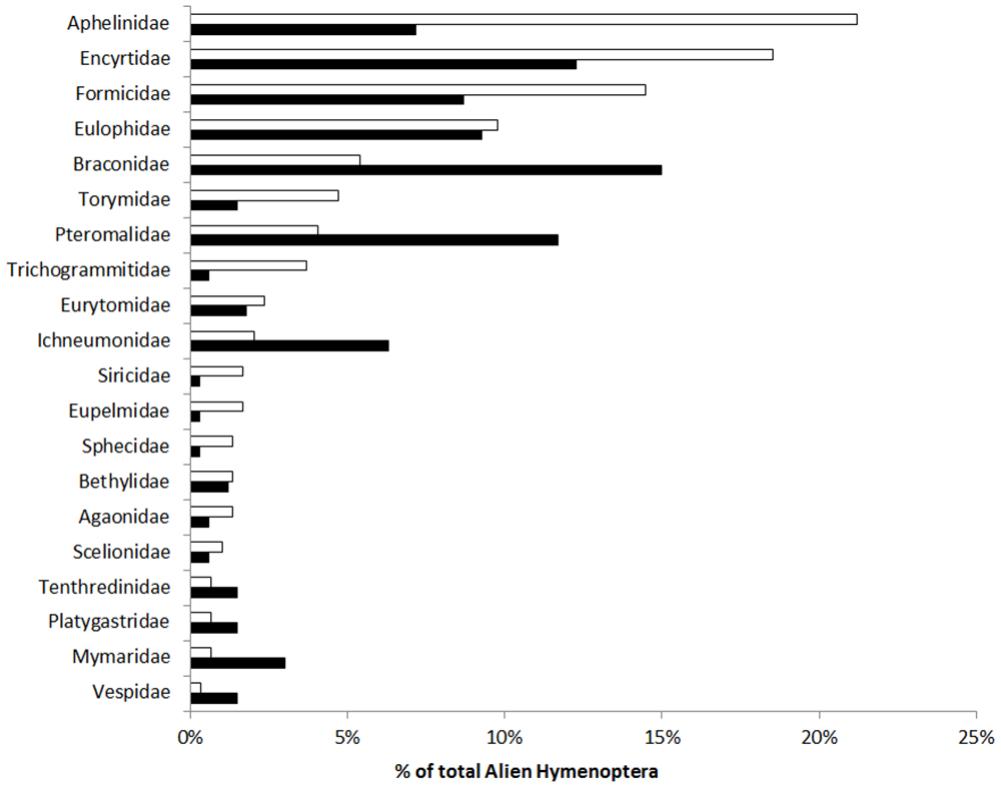
Comparison of alien Hymenoptera in Europe and New Zealand. Families are presented in a decreasing order based on a percent of total alien species from Europe. White = Europe; black = New Zealand.

There is a broad correlation (ρ = 0.704) in the number of alien species from different families between New Zealand and Europe; and where 6 of the top 7 families are the same (in New Zealand Ichneumonidae replaces Torymidae; Fig 8). The top 7 families make up a high proportion of the total alien species in both New Zealand (70%) and Europe (78%). However, the number of species alien to New Zealand is higher than for Europe (334 vs 286), and two crucial differences exist: i) the much lower number of intentionally released species in New Zealand (21% vs 63% in Europe); and ii) the greater proportion of parasitoids in New Zealand (71.2% vs 22.6%) amongst the unintentionally introduced species.

What is also striking is the proportion of alien species compared with the native fauna. For Europe, there are nine families where the number of alien species exceeds 5% of the native species known in Europe [31], but for New Zealand, this criterion is reached in almost all families (37 of 39 families), and in fact, 14 families have no native representatives.

As in Europe, alien Hymenoptera species are not evenly distributed throughout New Zealand and large differences exist between regions. However, a much larger proportion of alien species are found in urban areas in New Zealand (60.8%) compared with Europe (~30%, including houses, parks, green houses).

Both Europe and New Zealand have seen an exponential increase in the number of alien Hymenoptera during the last 150–200 years. However, unlike Europe where large increases were predominantly post-1950, there was also a large number of alien species present in early periods in New Zealand (e.g. from 1915 to 1950).

While North America (35.3%) and Asia (30.9%) provided the greatest part of alien Hymenoptera in Europe, this was not the case for New Zealand, where Australasia provided the greatest number of unintentional alien Hymenoptera (38.0%), followed by the Holarctic region (29.1%) and cosmopolitan species (24.0%). Unlike Europe, New Zealand data reveal a significant change in the origins of alien species over time, with an increasing dominance of Australasia during the past 25 years.

## Discussion

Developing generalisations of invasive species is an important part of invasion biology. In this study we presented the first inventory and analyses of all Hymenoptera alien to New Zealand, and by utilising information from the DAISIE project (Delivering Alien Invasive Species Inventories for Europe [31]), we compared two regions which in many respects are very different (e.g. geopolitics, trade volumes, land area). We asked: are trends and generalisations in alien Hymenoptera from one part of the world (e.g. Europe) consistent elsewhere (e.g. New Zealand). The answer is generally no, with few similarities evident.

One similarity was that families with the most species (e.g. Aphelinidae, Braconidae, Encyrtidae, Pteromalidae, Eulophidae, and Formicidae) were common for both regions, suggesting these families have some affinity for being invasive, or are more associated with human activities.

However, there were major differences in the alien Hymenoptera fauna between Europe and New Zealand, namely, the overall number of alien species; the proportion of unintentional introductions; the accumulation of species at different time periods; and the origins of alien species. Our study revealed that the number of alien Hymenoptera alien to New Zealand is higher than for Europe (334 vs 286). This is a considerable difference given the relatively small area size and trade of New Zealand compared to Europe. There is an even more striking difference when the numbers of intentionally released species are removed, and only numbers of unintentional introductions are compared: New Zealand (264) and Europe (106).

We are unsure why there is such a high disparity in the number of unintentionally introduced taxa between Europe and New Zealand, but suggest for New Zealand there are three filters mainly responsible for the current set of taxa: i) European settlement in the 1800s and early 1990s; ii) Australia as a regional source of alien species, especially in recent times; and iii) the importation of plants.

### European settlement

European settlement (mainly from the United Kingdom) has played an important part in forming the current set of alien species in New Zealand. From the mid-1800s to the early-1900s it is likely that many new species arrived unintentionally with the arrival of goods (e.g. machinery), stock, and crops associated with the new agriculture systems.

The introduction and impacts of alien vertebrates brought into New Zealand during European settlement have been well documented [24]. For weeds, it is also well recognised that during times of early settlement, dramatic human population growth and increased propagule pressure increased the likelihood of a plant species becoming invasive, in both Australia and New Zealand where there is a very similar history of European settlement [47–48]. Retrospective data shows that during these times, intentional introductions of plants (e.g. for food, fodder) were very prominent, and outnumbered unintentional introductions by a ratio 4:1 [47].

Although there has been comparatively less study, early European settlement is a well-known source of pest insects currently in New Zealand, including Hymenopteran species. Particularly well studied examples are the Cherry slug, *Caliroa cerasi* (Tenthredinidae), and the Sirex wood wasp, *Sirex noctilio* (Siricidae). The number of overall alien Hymenoptera species recorded during this period is very low (~4% of the total species presently known; Fig 4a). It is only from the 1920s onwards, that a significant increase in scientific effort (e.g. the formation of a dedicated national entomological collection and staff, faunal surveys, and a large increase in research associated with biological control of insect pests) led to a larger number of alien Hymenoptera being first recorded. Thus, while we consider that early settlement was an important time for the arrival and establishment of many alien Hymenoptera, the first reporting of many of these species occurred much later. This is an example of the ‘lag phase in reporting’ where there are big differences between the date of first record and the date of actual establishment.

### Regional sources of alien species

Biogeographic regions an important filter for invasive species. Regions which are geographically close are likely to be more climatically similar, and contain phylogenetically similar flora and faunas, thus allowing an invasive species to more easily become established and spread [47]. A number of species are known to have spread into neighbouring regions following their initial establishment. For example, the chestnut gall wasp *Dryocosmus kuriphilus* (Cynipidae) was introduced from China to Italy, and is now spreading unaided to neighbouring countries [31]. A recent country-level analysis of pests and diseases of woody plants showed distinct differences between northern, central and southern Europe in terms of their pest and pathogen assemblages due, at least in part, by spread within counties in these regions [49]. However, regions can also be important sources of invasive species through long-distance trade. For example, alien ants in the United States are heavily tied with the Neotropics [4], and China is an emerging source of new Hymenoptera species for Europe [31].

Overall, Australia was the source of the greatest number of unintentional alien Hymenoptera to New Zealand (98 species, 38.0%). This is somewhat surprising, given the current belief that Europe is the principal origin of large numbers of the alien flora and fauna in New Zealand. However, this belief is perhaps now only representative for the period of early settlement, as we found evidence of a recent shift towards Australia as the main origin for alien Hymenoptera for New Zealand.

Australia has previously been recognised as a source for the majority of alien ants in New Zealand [50], but recent alien Hymenoptera species from Australia show many different taxonomic groups are involved, for example: *Pison ruficorne* (Crabronidae), *Euryglossina hypochroma* (Colletidae), *Radumeris tasmaniensis* (Scoliidae), *Pleistodontes* fig wasps (Agaonidae) and various parasitoid groups [51–54]. Australia may have also acted as a secondary source of alien species, for example, there is strong evidence of Argentine ants being unintentionally introduced into New Zealand from Australia, although their ultimate origin is the Neotropics [27].

### Importation of plants

The importation of plants into a country may unintentionally bring in species which either i) directly feed on the plants (i.e. phytophagous species), or ii) that are associated with phytophagous species (i.e. predators, parasitoids). It is well recognised that the intentional importation of live plants, via horticultural, ornamental, and forestry pathways, is of major historical importance for the associated, but unintentional, introduction of pests and diseases [10, 15, 49]. National and international phytosanitary regulations have been developed to mitigate the unintentional introduction of other pests with plants. However, there are inconsistencies in the regulations and inspection intensity between countries, and in general such regulations are slow to respond to the continued trade of plants around the world [55].

Introductions of plants for planting and plant seeds are one of the main pathways of introduction for phytophagous Hymenoptera in Europe [31]. The seed pathway in particular appears to have been given little attention despite the repeated establishment of alien seed chalcid species in Europe [31]. Although there are relatively few unintentionally introduced phytophagous Hymenoptera in New Zealand (6 Symphyta [leaf miners and wood borer], 1 cynipid [gall former], and 16 chalcids [gall formers and seed predators]), a number of these species are significant economic pests. Several of which have been serious enough to have warranted biocontrol control programs against them; *Caliroa cerasi*, *Sirex noctilio*, and *Phylacteophaga froggatti* (Pergidae). None of these species attack native New Zealand species, rather they all feed upon intentionally introduced plants, for example, in plantation forestry (pine and Eucalypt plantations); *Salix* (willow) species for ornamental and riverside stabilisation (e.g. *Nematus oligospilus*), and horticulture orchards (e.g. *Caliroa cerasi*) [11, 56].

However, the importation of plants may also bring species that are associated with phytophagous species (i.e. their predators, parasitoids). One similarity between the New Zealand and European datasets was that several speciose families were common for both regions, suggesting those families have some affinity for being invasive, or are more associated with human activities. In particular, species of Aphelinidae, Encyrtidae, and Eulophidae are associated with plant-feeding hosts (especially Hemiptera), which would allow them to also be accidentally introduced when the host plant (and host insect) are imported. New Zealand border records show these three families have a very low proportion of being intercepted at the border but contribute a high proportion of established species. This suggests that these parasitoids group are less often discovered during border inspections, possibly because they are very small and have cryptic behaviours, including being inside their hosts.

### Conclusions

Hymenoptera are often well represented in studies of the impacts alien invertebrate [8], particularly ants (Formicidae) [22] and some predatory Hymenoptera [12,14]. Although ants were one of the families with a high number of alien species in New Zealand, several parasitoids groups had a similar, or higher, number of species. Despite a considerable amount of research in New Zealand on invasive social *Vespula* [25,26], and ants [57], our results show that greater attention should also be directed at the impacts of alien parasitoids, particularly as such a high proportion of accidentally introduced alien Hymenoptera are parasitoids in New Zealand (71.2%) compared with Europe (22.6%).

In our analyses we examined patterns associated with more than 330 alien Hymenoptera species currently established in New Zealand. European settlement, Australia as a regional source of alien species, and the importation of live plants, have acted as important invasion filters that have contributed a large invasion debt for New Zealand [5]. Few similarities exist with the alien Hymenoptera fauna introduced to Europe.

Differences in the composition of regional alien faunas are driven by a complex set of filters such as biogeography, socio-economic issues, global trade dynamics, and human populations [4–7]. Furthermore, the increasing amount, and efficiency, of global trade continues to provide the opportunity for new species to invade new areas [15]. Thus, obtaining generalisations and predictions can be difficult, especially if there is only a limited set of taxa and regions available [58]. Teasing apart patterns of invasive species and trade related statistics (e.g. different commodity types, agriculture, vs non-agriculture etc) is important. However, examining broad taxonomic patterns is often a useful starting point for understanding invasions [15, 48].

We recommend that further effort be made towards the formation, and analysis, of regional inventories of alien species. This will allow a wider range of taxa and regions to be examined for generalisations, and help assess the risk posed by certain taxa to the economy or environment, and which habitats are at greater risk.

## Supporting Information

S1 Table List of Hymenoptera species unintentionally introduced to New Zealand. “1st year” is the date a species was first recorded as established in New Zealand. “#Invaded regions” refers to area codes in Crosby et al. (1998, maximum number of regions = 28); cells with an ‘x’ were considered to be poorly known (based on collection records) and were not analysed. “Additional sources” were used to supplement information from Gordon et al. (2010) and specimen records in the New Zealand Arthropod Collection. Last update 31/12/2014.
(DOCX)

S2 Table List of Hymenoptera species intentionally released into New Zealand. “1st year” is the date a species was first recorded as established in New Zealand. “Additional sources” were used to supplement information from Ferguson et al. (2007), Gordon et al. (2010) and specimen records in the New Zealand Arthropod Collection. Last update 31/12/2014.
(DOCX)
