# Supplementary material for: Poles Apart: Comparing Trends of Alien Hymenoptera in New Zealand with Europe (DAISIE)
Source: PLoS One. 2015 Jul 6;10(7):e0132264. doi: 10.1371/journal.pone.0132264 (PMC4492945; doi:10.1371/journal.pone.0132264)
Supplement: S1 Table — “1st year” is the date a species was first recorded as established in New Zealand. “#Invaded regions” refers to area codes in Crosby et al. (1998, maximum number of regions = 28); cells with an ‘x’ were considered to be poorly known (based on collection records) and were not analysed. “Additional sources” were used to supplement information from Gordon et al. (2010) and specimen records in the New Zealand Arthropod Collection. Last update 31/12/2014. (DOCX) [file pone.0132264.s001.docx]

**Supporting Information Table S1. List of Hymenoptera species unintentionally introduced to New Zealand.** “1^st^ year” is the date a species was first recorded as established in New Zealand. “#Invaded regions” refers to area codes in Crosby et al. (1998, maximum number of regions = 28); cells with an ‘x’ were considered to be poorly known (based on collection records) and were not analysed. “Additional sources” were used to supplement information from Gordon et al. (2010) and specimen records in the New Zealand Arthropod Collection. Last update 31/12/2014.

| **Family**  **Genus + Species** | **Functional**  **Group** | **Origin** | **Host** | **1st Year** | **#Invaded Regions** | **Key Sources** |
| --- | --- | --- | --- | --- | --- | --- |
| **Agaonidae** |  |  |  |  |  |  |
| *Pleistodontes froggatti* Mayr, 1906 | pollinator | Australasia | *Ficus* | 1993 | 7 | Early 2000; Gardner & Early 1996 |
| *Pleistodontes imperialis* Saunders, 1883 | pollinator | Australasia | *Ficus* | 1972 | 5 | Early 2000; Gardner & Early 1996 |
| **Aphelinidae** |  |  |  |  |  |  |
| *Aphelinus abdominalis* (Dalman, 1820) | parasitoid | Cosmopolitan | Hemiptera | 1943 | x |  |
| *Aphelinus asychis* Walker, 1839 | parasitoid | Cosmopolitan | Hemiptera | 1957 | x |  |
| *Aphelinus gossypii* Timberlake, 1924 | parasitoid | Cosmopolitan | Hemiptera | 1930 | x |  |
| *Aphelinus humilis* Mercet, 1928 | parasitoid | Palaearctic | Hemiptera | 1971 | x |  |
| *Aphytis chilensis* Howard, 1900 | parasitoid | Cosmopolitan | Hemiptera | 1962 | x |  |
| *Aphytis chrysomphali* (Mercet, 1912) | parasitoid | Cosmopolitan | Hemiptera | 1962 | x |  |
| *Aphytis diasidis* (Howard, 1881) | parasitoid | Cosmopolitan | Hemiptera | 1935 | x |  |
| *Aphytis ignotus* Compere, 1955 | parasitoid | Australasia | Hemiptera | 1962 | x |  |
| *Aphytis mytilaspidis* (Le Baron, 1870) | parasitoid | Cosmopolitan | Hemiptera | 1935 | x | Valentine 1975 |
| *Centrodora scolypopae* Vallentine, 1966 | parasitoid | Australasia | Hemiptera | 1961 | x |  |
| *Centrodora xiphidii* (Perkins, 1906) | parasitoid | Neotropical | Orthoptera | unknown | x |  |
| *Coccophagus philippiae* (Silvestri, 1915) | parasitoid | Africa | Hemiptera | 1962 | x |  |
| *Coccophagus scutellaris* (Dalman, 1825) | parasitoid | Cosmopolitan | Hemiptera | 1962 | x |  |
| *Encarsia citrina* (Craw, 1891) | parasitoid | Cosmopolitan | Hemiptera | 1923 | 2 |  |
| *Encarsia inaron* (Walker, 1839) | parasitoid | Cosmopolitan | Hemiptera | 1997 | x | Charles 1998 |
| *Encarsia koebelei* (Howard, 1908) | parasitoid | Nearctic | Hemiptera | 1989 | x |  |
| *Encarsia pergandiella* Howard, 1907 | parasitoid | Cosmopolitan | Hemiptera | 1975 | x |  |
| *Encarsia perniciosi* (Tower, 1913) | parasitoid | Cosmopolitan | Hemiptera | 1959 | x |  |
| *Eretmocerus warrae*  Naumann & Schmidt, 2000 | parasitoid | Australasia | Hemiptera | 1997 | x |  |
| **Bethylidae** |  |  |  |  |  |  |
| *Chilepyris platythelys* Sorg & Walker, 1989 | parasitoid | Australasia | Coleoptera | 1980 | 2 | Ward 2013 |
| *Goniozus jacintae* Farrugia, 1981 | parasitoid | Australasia | Lepidoptera | 1922 | 8 | Ward 2013 |
| *Plastanoxus laevis* (Ashmead, 1893) | parasitoid | Cosmopolitan | Coleoptera | 1925 | 3 | Ward 2013 |
| *Sclerodermus niveifemur* (Evans, 1964) | parasitoid | Australasia | Coleoptera | 1967 | 3 | Ward 2013 |
| **Braconidae** |  |  |  |  |  |  |
| *Apanteles carpatus* (Say, 1836) | parasitoid | Cosmopolitan | Lepidoptera | 1948 | 2 |  |
| *Apanteles galleriae* Wilkinson, 1932 | parasitoid | Cosmopolitan | Lepidoptera | 1980 | 1 |  |
| *Aphaereta pallipes* (Say, 1829) | parasitoid | Australasia | Diptera | 1980 | 1 | Berry 2007a |
| *Aphidius colemani* Viereck, 1912 | parasitoid | Oriental | Hemiptera | 1976 | 3 |  |
| *Aphidius pelargonii* Stary & Carver, 1979 | parasitoid | Australasia | Hemiptera | 1930 | 2 |  |
| *Aphidius salicis* Haliday, 1834 | parasitoid | Palaearctic | Hemiptera | 1983 | 1 |  |
| *Aphidius similis* Stary & Carver, 1979 | parasitoid | Australasia | Hemiptera | 1981 | 1 |  |
| *Aphidius sonchi* Marshall, 1896 | parasitoid | Palaearctic | Hemiptera | 1996 | 2 |  |
| *Aridelus rufotestaceus* Tobias, 1986 | parasitoid | Holarctic | Hemiptera | 2013 | 1 |  |
| *Asobara persimilis* (Prince, 1976) | parasitoid | Australasia | Diptera | 1941 | 7 | Berry 2007a |
| *Asobara tabida* (Nees von Esenbeck, 1834) | parasitoid | Europe | Diptera | 1996 | 1 | Berry 2007a |
| *Bracon hebetor* (Say, 1836) | parasitoid | Palaearctic | Lepidoptera | 1924 | 7 | Parrott 1954 |
| *Chremylus elaphus* Haliday, 1833 | parasitoid | Cosmopolitan | Lepidoptera | 1948 | 2 |  |
| *Cotesia ruficrus* (Haliday, 1834) | parasitoid | unknown | Lepidoptera | 1921 | 15 |  |
| *Dacnusa areolaris* (Nees, 1812) | parasitoid | Europe | Diptera | 1920 | 11 | Berry 2007a |
| *Diaeretiella rapae* (M’Intosh, 1855) | parasitoid | Cosmopolitan | Hemiptera | 1921 | 8 | Todd 1957 |
| *Dinocampus coccinellae* (Shrank, 1802) | parasitoid | Cosmopolitan | Coleoptera | 1921 | 9 |  |
| *Dolichogenidea tasmanica* (Cameron, 1912) | parasitoid | Australasia | Lepidoptera | 1921 | 3 | Valentine & Walker 1991 |
| *Lysiphlebus testaceipes* (Cresson, 1880) | parasitoid | Holarctic | Hemiptera | 1977 | 4 |  |
| *Meteorus cespitator* (Thunberg, 1822) | parasitoid | Palaearctic | Lepidoptera | 1927 | 2 | Huddleston 1986 |
| *Meteorus cinctellus* (Spinola, 1808) | parasitoid | Palaearctic | Lepidoptera | 1906 | 12 | Huddleston 1986 |
| *Meteorus pulchricornis* (Wesmael, 1835) | parasitoid | Palaearctic | Lepidoptera | 1996 | 8 | Berry 1997a |
| *Monolexis fuscicornis* Förster, 1862 | parasitoid | Cosmopolitan | Coleoptera | 1942 | 1 | Belokobylskij et al. 2004 |
| *Ontsira antica* (Wollaston, 1858) | parasitoid | Holarctic | Coleoptera | 1950 | 1 |  |
| *Opius cinerariae* Fisher, 1963 | parasitoid | Australasia | Diptera | 1996 | 8 |  |
| *Parallorhogas pallidiceps* (Perkins, 1910) | parasitoid | Oriental | Coleoptera | unknown | x |  |
| *Pseudochremylus angulifer*  van Achterberg, 2005 | parasitoid | Australasia | unknown | 1981 | 1 |  |
| *Spathius exarator* (Linnaeus, 1758) | parasitoid | Palaearctic | Coleoptera | 1953 | 3 |  |
| *Spathius pedestris* Wesmael, 1838 | parasitoid | Europe | Coleoptera | 1953 | 4 | Belokobylskij & Austin 2013 |
| **Chalcididae** |  |  |  |  |  |  |
| *Brachymeria rubrifemur* (Girault, 1913) | parasitoid | Australasia | Lepidoptera | 1969 | x |  |
| **Colletidae** |  |  |  |  |  |  |
| *Euryglossina hypochroma* Cockerell, 1916 | pollinator | Australasia | Myrtaceae | 2006 | 1 | Donovan 2007 |
| *Euryglossina proctotrypoides*  Cockerell, 1913 | pollinator | Australasia | Myrtaceae | 1942 | 5 | Donovan 2007 |
| *Hylaeus asperithorax* (Rayment, 1927) | pollinator | Australasia | numerous | 1929 | 12 | Donovan 2007 |
| *Hylaeus perhumilis* (Cockerell, 1914) | pollinator | Australasia | mostly Myrtaceae | 1973 | 3 | Donovan 2007 |
| *Hyleoides concinna* (Fabricius, 1775) | pollinator | Australasia | numerous | 1980 | 10 | Donovan 2007 |
| **Crabronidae** |  |  |  |  |  |  |
| *Pison ruficorne* Smith, 1856 | predator | Australasia | Araneae | 2007 | 1 |  |
| *Pison spinolae* Shuckard, 1837 | predator | Australasia | Araneae | 1880 | 28 | McC Callan 1979 |
| **Cynipidae** |  |  |  |  |  |  |
| *Phanacis hypochoeridis* (Keifer, 1887) | phytophagous-  gall former | Cosmopolitan | *Hypochaeris* | 1922 | 9 | Ward 2014 |
| **Diapriidae** |  |  |  |  |  |  |
| *Neurogalesus carinatus* Kieffer, 1907 | parasitoid | Australasia | Diptera | 1985 | 4 |  |
| *Neurogalesus militis*  Osborn, Forteath & Holloway, 1973 | parasitoid | Australasia | Diptera | 2012 | 1 |  |
| **Dryinidae** |  |  |  |  |  |  |
| *Dryinus koebelei* (Perkins, 1905) | parasitoid | Australasia | Hemiptera | 2004 | 1 | Olmi 2007 |
| **Encyrtidae** |  |  |  |  |  |  |
| *Adelencyrtus aulacaspidis* (Brethes, 1914) | parasitoid | Palaearctic | Hemiptera | 1962 | 3 | Charles 1998 |
| *Alamella mira* Noyes, 1988 | parasitoid | Australasia | Hemiptera | 1981 | 1 |  |
| *Anagyrus fusciventris* (Girault, 1915) | parasitoid | Australasia | Hemiptera | 1991 | 1 |  |
| *Arrhenophagoidea coloripes* Girault, 1915 | parasitoid | Australasia | Hemiptera | 1962 | 1 |  |
| *Arrhenophagus chionaspidis*  Aurivillius, 1888 | parasitoid | Cosmopolitan | Hemiptera | 1962 | x |  |
| *Baeoanusia albifunicle* Girault, 1932 | parasitoid | Australasia | Hymenoptera | 2005 | 1 |  |
| *Cheiloneurus flaccus* (Walker, 1847) | parasitoid | Neotropical | Hemiptera | 2013 | 1 | Thorpe 2013a |
| *Coccidoctonus psyllae* (Riek, 1962) | parasitoid | Australasia | Hemiptera | 2006 | 2 | Berry 2007b |
| *Cryptanusia aureiscutellum* (Girault, 1926) | parasitoid | Australasia | Hemiptera | unknown | x |  |
| *Encyrtus aurantii* (Geoffroy, 1785) | parasitoid | Cosmopolitan | Hemiptera | 1962 | 1 |  |
| *Encyrtus infelix* (Embleton, 1902) | parasitoid | Africa | Hemiptera | 1928 | 2 |  |
| *Epiblatticida minutissima* (Girault, 1923) | parasitoid | Australasia | Hymenoptera | 1980 | 1 |  |
| *Epitetracnemus intersectus*  (Fonscolombe, 1832) | parasitoid | Holarctic | Hemiptera | 1960 | x |  |
| *Eusemion cornigerum* (Walker, 1838) | parasitoid | Palaearctic | Hemiptera | 1978 | 5 |  |
| *Gyranusoidea advena* Beardsley, 1969 | parasitoid | Nearctic | Hemiptera | 1962 | 1 |  |
| *Lamennaisia ambigua* (Nees, 1834) | parasitoid | Palaearctic | Coleoptera | 1959 | 8 |  |
| *Metanotalia maderensis* (Walker, 1872) | parasitoid | Europe | Hemiptera | 1970 | 1 | Noyes 1988 |
| *Metaphycus alberti* (Howard, 1898) | parasitoid | Cosmopolitan | Hemiptera | 1926 | x |  |
| *Metaphycus anneckei*  Guerrieri & Noyes, 2000 | parasitoid | Africa | Hemiptera | unknown | x | Guerrieri & Noyes 2000 |
| *Metaphycus maculipennis* (Timberlake, 1916) | parasitoid | Nearctic | Hemiptera | 1951 | 1 |  |
| *Psyllaephagus acaciae* Noyes, 1988 | parasitoid | Australasia | Hemiptera | 1927 | 2 | Noyes 1988 |
| *Psyllaephagus bliteus* Riek, 1962 | parasitoid | Australasia | Hemiptera | 2000 | 2 | Noyes 1988 |
| *Psyllaephagus breviramus* Berry, 2007 | parasitoid | Australasia | Hemiptera | 1971 | 2 | Berry 2007b |
| *Psyllaephagus cornwallensis* Berry, 2007 | parasitoid | Australasia | Hemiptera | 2000 | 1 | Berry 2007b |
| *Psyllaephagus gemitus* Riek, 1962 | parasitoid | Australasia | Hemiptera | 1999 | 2 | Noyes 1988 |
| *Psyllaephagus pilosus* Noyes, 1988 | parasitoid | Australasia | Hemiptera | 1925 | 3 | Noyes 1988 |
| *Psyllaephagus richardhenryi* Berry, 2007 | parasitoid | Australasia | Hemiptera | 2002 | 1 | Berry 2007b |
| *Rhopus garibaldia* (Girault, 1933) | parasitoid | Australasia | Hemiptera | 1957 | 1 |  |
| *Syrphophagus aphidivorus* (Mayr, 1876) | parasitoid | Cosmopolitan | Several orders | 2004 | 1 |  |
| *Tachinaephagus australiensis* (Girault, 1914) | parasitoid | Australasia | Diptera | unknown | x | Thompson 1955 |
| *Tetracnemoidea peregrine* (Compere, 1939) | parasitoid | Cosmopolitan | Hemiptera | 1961 | 1 |  |
| *Tetracnemoidea sydneyensis*  (Timberlake, 1929) | parasitoid | Cosmopolitan | Hemiptera | 1962 | 1 |  |
| *Zaomma lambinus* (Walker, 1838) | parasitoid | Cosmopolitan | Hemiptera | 1960 | 1 |  |
| **Eulophidae** |  |  |  |  |  |  |
| *Aprostocetus zosimus* (Walker, 1839) | parasitoid | Nearctic | Diptera | 1959 | x | Cumber 1959 |
| *Baryscapus bruchophagi* (Gahan, 1913) | parasitoid | Nearctic | Several orders | 1927 | 3 |  |
| *Baryscapus galactopus* (Ratzeburg, 1844) | parasitoid | Nearctic | Several orders | 1990 | 2 |  |
| *Ceranisius menes* (Walker, 1839) | parasitoid | Cosmopolitan | Thysanoptera | 1967 | 1 |  |
| *Chrysocharis gemma* (Walker, 1839) | parasitoid | Palaearctic | Several orders | unknown | x | Hansson 1985 |
| *Chrysocharis pubicornis* (Zetterstedt, 1838) | parasitoid | Palaearctic | Several orders | 1948 | 1 |  |
| *Cirrospilus vittatus* Walker, 1838 | parasitoid | Cosmopolitan | Several orders | 2002 | 1 |  |
| *Closterocerus cruy* (Girault, 1918) | parasitoid | Australasia | Lepidoptera | 1927 | 4 |  |
| *Diglyphus isaea* (Walker, 1838) | parasitoid | Cosmopolitan | Diptera | 1975 | 1 |  |
| *Entedon methion* Walker, 1839 | parasitoid | Holarctic | Coleoptera | 1946 | 4 |  |
| *Hemiptarsenus varicornis* (Girault, 1913) | parasitoid | Cosmopolitan | Diptera | 1924 | 1 |  |
| *Melittobia acasta* (Walker, 1839) | parasitoid | Cosmopolitan | Several orders | 1987 | 1 |  |
| *Melittobia australica* Girault, 1912 | parasitoid | Cosmopolitan | Several orders | 1980 | 1 |  |
| *Melittobia hawaiiensis* Perkins, 1907 | parasitoid | Cosmopolitan | Several orders | unknown | x |  |
| *Neochrysocharis formosus*  (Westwood, 1833) | parasitoid | Cosmopolitan | Several orders | 1989 | 1 |  |
| *Neochrysocharis trifolii* Erdos, 1961 | parasitoid | Europe | Lepidoptera | 1977 | x |  |
| *Neotrichoporoides viridimaculatus*  (Fullaway, 1955) | parasitoid | Palaearctic | Lepidoptera | 2003 | 1 |  |
| *Oomyzus scaposus* (Thompson, 1878) | parasitoid | Cosmopolitan | Coleoptera | unknown | x |  |
| *Ophelimus eucalypti* (Gahan, 1922) | phytophagous-  gall former | Australasia | *Eucalyptus* | 1924 | 10 | Bain 1977a; Withers 2001 |
| *Ophelimus maskelli* (Ashmead, 1900) | phytophagous-  gall former | Australasia | *Eucalyptus* | 1961 | 3 | Noyes 2014 |
| *Pediobius bruchicida* (Rondani, 1872) | parasitoid | Palaearctic | Several orders | 1922 | 3 |  |
| *Pnigalio pectinicornis* (Linnaeus, 1758) | parasitoid | Palaearctic | Several orders | 1955 | 2 | Swan 1973; Valentine & Walker 1991 |
| *Pnigalio soemius* (Walker, 1839) | parasitoid | Palaearctic | Several orders | 1952 | 4 | Hill et al. 2001 |
| *Quadrastichodella aena* Girault, 1913 | phytophagous-  gall former | Australasia | *Eucalyptus* | unknown | x | Kim Il-Kwon et al 2005 |
| *Quadrastichodella nova* Girault, 1922 | phytophagous-  gall former | Australasia | *Eucalyptus* | 1962 | x | Kim Il-Kwon et al 2005 |
| *Quadrastichodella pilosa* Ikeda, 1999 | phytophagous-  gall former | Australasia | *Eucalyptus* | unknown | x | Kim Il-Kwon et al 2005 |
| *Sympiesis sericeicornis* (Nees, 1834) | parasitoid | Cosmopolitan | Several orders | 2003 | 1 |  |
| **Eupelmidae** |  |  |  |  |  |  |
| *Eupelmus vesicularis* (Ratzius, 1783) | parasitoid | Holarctic | numerous | 1964 | x |  |
| **Eurytomidae** |  |  |  |  |  |  |
| *Bruchophagus acacia* (Cameron, 1910) | phytophagous-  seed predator | Australasia | *Acacia* | 1910 | 2 | Valentine & Walker 1991; Hill et al 2000 |
| *Bruchophagus gibbus* (Boheman, 1836) | phytophagous-  seed predator | Europe | (Leguminosae) *Trifolium* | 1921 | 3 | Valentine 1970 |
| *Bruchophagus roddi* (Gussakovsky, 1933) | phytophagous-  seed predator | Cosmopolitan | (Leguminosae) Medicago, including lucerne | 1962 | 1 | Valentine 1970 |
| *Dougiola* sp. | phytophagous-  seed predator | Australasia | *Casuarina* | 2013 | 1 |  |
| *Systole foeniculi* Otten, 1941 | phytophagous-  seed predator | Europe | *Conium maculatum* and *Foeniculum officinale* | 1924 | 2 | Valentine 1967; Gourlay 1930 |
| *Tetramesa linearis* (Walker, 1832) | phytophagous-  gall former | Europe | *Agropyron repens* (Gramineae) | 1943 | 4 | Goolsby & Moran 2009 |
| **Figitidae** |  |  |  |  |  |  |
| *Alloxysta fuscicornis* (Hartig, 1841) | parasitoid | Cosmopolitan | Hymenoptera | 1927 | 5 | Ward 2014 |
| *Alloxysta rubidus*  Ferrer-Suay & Pujade-Villar, 2012 | parasitoid | Australasia | Hymenoptera | 1966 | 9 | Ferrer-Suay et al 2012 |
| *Alloxysta thorpei*  Ferrer-Suay & Pujade-Villar, 2012 | parasitoid | Australasia | Hymenoptera | 1924 | 11 | Ferrer-Suay et al 2012 |
| *Alloxysta victrix* Westwood, 1833 | parasitoid | Cosmopolitan | Hymenoptera | 1927 | 12 | Ward 2014 |
| *Ganaspis* sp. | parasitoid | unknown | Diptera | 1990 | 2 | Ward 2014 |
| *Hexacola* sp. | parasitoid | unknown | Diptera | 1929 | 8 | Ward 2014 |
| *Leptopilina heterotoma* (Thomson, 1862) | parasitoid | unknown | Diptera | 1974 | 3 | Ward 2014 |
| *Phaenoglyphis villosa* (Hartig, 1841) | parasitoid | Cosmopolitan | Hymenoptera | 1927 | 8 | Ward 2014 |
| *Thoreauella* sp. | parasitoid | unknown | Diptera | 1957 | 6 | Ward 2014 |
| *Trybliographa* sp. | parasitoid | Nearctic | Diptera | 1926 | 7 | Ward 2014 |
| *Xyalaspis* sp. | parasitoid | unknown | Neuroptera | 1959 | 6 | Ward 2014 |
| **Formicidae** |  |  |  |  |  |  |
| *Amblyopone australis* Erichson, 1842 | predator | Australasia | numerous | 1876 | 13 | Don 2007; Ward 2005 |
| *Cardiocondyla minutior* Forel, 1899 | predator | Neotropical | numerous | 2000 | 2 | Harris & Berry 2001 |
| *Doleromyrma darwiniana* (Forel, 1907) | predator | Australasia | numerous | 1959 | 9 | Don 2007; Ward 2005 |
| *Hypoponera confinis* (Roger, 1860) | predator | Oriental | numerous | 2007 | 1 |  |
| *Hypoponera eduardi* (Forel, 1894) | predator | Europe | numerous | 1947 | 1 | Don 2007; Ward 2005 |
| *Hypoponera punctatissima* (Roger, 1859) | predator | Palaearctic | numerous | 2003 | 20 | Don 2007; Ward 2005 |
| *Iridomyrmex suchieri* Forel, 1907 | predator | Australasia | numerous | 1954 | 9 | Don 2007; Ward 2005 |
| *Linepithema humile* (Mayr, 1868) | predator | Neotropical | numerous | 1990 | 13 | Don 2007; Ward 2005 |
| *Mayriella abstinens* Forel, 1902 | predator | Australasia | numerous | 1957 | 1 | Harris & Berry 2001 |
| *Monomorium pharaonis* (Linnaeus, 1758) | predator | Palaearctic | numerous | 1941 | 5 | Green 1992 |
| *Monomorium sydneyense* Forel, 1902 | predator | Australasia | numerous | 2003 | 2 | Don 2007; Ward 2005 |
| *Nylanderia braueri glabrior* (Forel, 1902) | predator | Australasia | numerous | unknown | x |  |
| *Nylanderia* sp. | predator | Australasia | numerous | unknown | x |  |
| *Nylanderia tasmaniensis* (Forel, 1913) | predator | Australasia | numerous | unknown | x |  |
| *Ochetellus glaber* (Mayr, 1862) | predator | Australasia | numerous | 1927 | 11 | Don 2007; Ward 2005 |
| *Orectognathus antennatus* Smith, 1853 | predator | Australasia | numerous | 1927 | 4 | Don 2007; Ward 2005 |
| *Pheidole megacephala* (Fabricius, 1793) | predator | Africa | numerous | 1942 | 1 | Don 2007; Ward 2005 |
| *Pheidole proxima* Mayr, 1876 | predator | Australasia | numerous | 2004 | 1 | Green & Gunawardana 2006 |
| *Pheidole rugosula* Forel, 1902 | predator | Australasia | numerous | 1958 | 9 | Don 2007; Ward 2005 |
| *Pheidole vigilans* (Smith, 1858) | predator | Australasia | numerous | 1949 | 3 | Don 2007; Ward 2005 |
| *Ponera leae* Forel, 1913 | predator | Australasia | numerous | 1958 | 3 | Harris & Berry 2001 |
| *Rhytidoponera chalybaea* Emery, 1901 | predator | Australasia | numerous | 1959 | 6 | Don 2007; Ward 2005 |
| *Rhytidoponera metallica* (Smith, 1858) | predator | Australasia | numerous | 1958 | 1 | Don 2007; Ward 2005 |
| *Solenopsis* sp. | predator | Australasia | numerous | 2001 | 1 | Don 2007; Ward 2005 |
| *Strumigenys perplexa* (Smith, 1876) | predator | Australasia | numerous | 1870 | 7 | Don 2007; Ward 2005 |
| *Strumigenys xenos* Brown, 1955 | predator | Australasia | numerous | 1959 | 5 | Don 2007; Ward 2005 |
| *Technomyrmex jocosus* Forel, 1910 | predator | Australasia | numerous | 1921 | 16 | Don 2007; Ward 2005 |
| *Tetramorium bicarinatum* (Nylander, 1846) | predator | Oriental | numerous | 1959 | 4 | Don 2007; Ward 2005 |
| *Tetramorium grassii* Emery, 1895 | predator | Africa | numerous | 1941 | 7 | Don 2007; Ward 2005 |
| **Halictidae** |  |  |  |  |  |  |
| *Lasioglossum cognatum* (Smith, 1853) | pollinator | Australasia | numerous | 1949 | 8 | Donovan 2007 |
| **Ichneumonidae** |  |  |  |  |  |  |
| *Ctenochares bicolorus* (Linnaeus, 1767) | parasitoid | Africa | Lepidoptera | 1981 | 1 | Fitton et al 1983 |
| *Diplazon laetatorius* (Fabricius, 1781) | parasitoid | Cosmopolitan | Diptera | 1878 | 22 | Smith 1878 |
| *Gelis cinctus* (Linnaeus, 1758) | parasitoid | Europe | Lepidoptera & Hymenoptera | 1922 | 9 | Russell 1987 |
| *Gelis tenellus* (Say, 1836) | parasitoid | Nearctic | Lepidoptera & Hymenoptera | 1963 | 6 | Russell 1987 |
| *Hypsicera femoralis* (Geoffroy, 1785) | parasitoid | Cosmopolitan | Lepidoptera | 1977 | 2 | Berry 1990 |
| *Oedemopsis* sp. | parasitoid | Australasia | Lepidoptera | 2011 | 1 |  |
| *Rhyssa lineolate* (Kirby, 1837) | parasitoid | Nearctic | Hymenoptera | 1958 | 5 | Bain et al 2012 |
| *Stenomacrus* sp. | parasitoid | Nearctic | Diptera | 1922 | 2 |  |
| *Venturia canescens* (Gravenhorst, 1829) | parasitoid | Cosmopolitan | Lepidoptera | 1978 | 4 | Waller 1982 |
| *Xenolytus bitinctus* (Gmelin, 1790) | parasitoid | Europe | Lepidoptera | 1953 | 1 |  |
| **Megachilidae** |  |  |  |  |  |  |
| *Anthidium manicatum* (Linnaeus, 1758) | pollinator | Europe | numerous | 2006 | 13 | Soper & Berry 2013 |
| **Megaspilidae** |  |  |  |  |  |  |
| *Dendrocerus aphidum* (Rondani, 1877) | parasitoid | Cosmopolitan | Hymenoptera | 1959 | 5 |  |
| *Dendrocerus carpenteri* (Curtis, 1829) | parasitoid | Cosmopolitan | Hymenoptera | 1921 | 8 | Todd 1957 |
| *Dendrocerus laticeps* (Hedicke, 1929) | parasitoid | Cosmopolitan | Hymenoptera | unknown | 4 |  |
| *Trichosteresis glaber* (Boheman, 1832) | parasitoid | Palaearctic | unknown | 1977 | 4 |  |
| **Mutillidae** |  |  |  |  |  |  |
| *Ephutomorpha bivulnerata* (André, 1901) | parasitoid | Australasia | Hymenoptera | 1963 | 2 | Valentine & Walker 1983 |
| **Mymaridae** |  |  |  |  |  |  |
| *Anagrus atomus* (Linnaeus, 1767) | parasitoid | Palaearctic | Hemiptera | 1978 | 5 |  |
| *Anagrus avalae* Soyka, 1955 | parasitoid | Palaearctic | Hemiptera | 1980 | 1 |  |
| *Anagrus frequens* Perkins, 1905 | parasitoid | Oriental | Hemiptera | 1960 | 2 | Triapitsyn 2001 |
| *Anagrus incarnatus* Haliday, 1833 | parasitoid | Palaearctic | Hemiptera | 1980 | 4 | Triapitsyn 2001 |
| *Anagrus optabilis* (Perkins, 1905) | parasitoid | Oriental | Hemiptera | 1981 | 2 | Triapitsyn 2001 |
| *Anagrus ustulatus* Haliday, 1833 | parasitoid | Palaearctic | Hemiptera | 1932 | 1 |  |
| *Mymar schwanni* Girault, 1912 | parasitoid | Australasia | Hemiptera | 1970 | 8 | Triapitsyn & Berezovskiy 2001 |
| *Mymar taprobanicum* Ward, 1875 | parasitoid | Cosmopolitan | Hemiptera | 1999 | x | Triapitsyn & Berezovskiy 2001 |
| *Stephanodes reduvioli* (Perkins, 1905) | parasitoid | Cosmopolitan | Hemiptera | 1959 | 2 | Huber & Fidalgo 1997 |
| **Pergidae** |  |  |  |  |  |  |
| *Phylacteophaga froggatti* Riek, 1955 | phytophagous-  leaf miner | Australasia | *Eucalyptus* | 1985 | 16 | Kay 1986 |
| **Platygastridae** |  |  |  |  |  |  |
| *Aphanomerus pusillus* Perkins, 1905 | parasitoid | Australasia | Hemiptera | 1922 | 5 | Gourlay 1930; Charles 1998 |
| *Fidiobia ?citri* (Nixon, 1969) | parasitoid | Neotropical | Coleoptera | 1976 | 8 | Charles 1998 |
| *Platygaster hiemalis* Forbes, 1888 | parasitoid | Europe | Diptera | 1893 | x | Valentine & Walker 1991 |
| *Probaryconus dubius* (Nixon, 1931) | parasitoid | Africa | Orthoptera | 1957 | 6 | Hill 1983 |
| **Pompilidae** |  |  |  |  |  |  |
| *Cryptocheilus australis* (Guérin, 1830) | predator | Australasia | Araneae | 1963 | 6 | Harris 1987 |
| **Proctotrupidae** |  |  |  |  |  |  |
| *Exallonyx trifoveatus* Kieffer, 1908 | parasitoid | Cosmopolitan | Coleoptera | 1941 | 2 | Valentine & Walker 1991; Townes & Townes 1981 |
| **Pteromalidae** |  |  |  |  |  |  |
| *Acroclisoides* sp. | parasitoid | Australasia | Hemiptera | 1995 | 2 |  |
| *Anisopteromalus calandrae* (Howard, 1881) | parasitoid | Cosmopolitan | Coleoptera | unknown | x |  |
| *Asaphes vulgaris* Walker, 1834 | parasitoid | Cosmopolitan | Hemiptera | 1987 | 4 |  |
| *Camarothorax* sp. | phytophagous? | Australasia | *Ficus* | 1993 | 1 |  |
| *Dibrachys microgastri* (Bouché, 1834) | parasitoid | Cosmopolitan | Several orders | 1995 | 3 | Peters & Baur 2011 |
| *Eukoebelea australiensis* (Ashmead, 1904) | phytophagous | Australasia | *Ficus* | 2013 | 1 |  |
| *Herodotia subatriventris* (Girault, 1923) | phytophagous | Australasia | *Ficus* | 1980 | 1 | Early 2000; Gardner & Early 1996 |
| *Homoporus nypsius* Walker, 1839 | parasitoid | Holarctic | Several orders | unknown | x |  |
| *Lariophagus distinguendus* (Förster, 1840) | parasitoid | Cosmopolitan | Coleoptera | 1923 | 4 |  |
| *Mesopolobus incultus* (Walker, 1834) | parasitoid | Europe | Coleoptera | unknown | x |  |
| *Mesopolobus nobilis* (Walker, 1834) | parasitoid | Europe | Coleoptera | unknown | x |  |
| *Moranila californica* (Howard, 1881) | parasitoid | Australasia | Hemiptera | 1960 | 2 | Berry 1995 |
| *Moranila comperei* (Ashmead, 1904) | parasitoid | Australasia | Hemiptera | 1994 | 1 | Berry 1995 |
| *Nambouria xanthops* Berry & Withers, 2002 | phytophagus-  gall former | Australasia | *Eucalyptus* | 1999 | 1 | Berry & Withers 2002 |
| *Neopolycystus insectifurax* Girault, 1915 | parasitoid | Australasia | Coleoptera | 2002 | 2 |  |
| *Notoglyptus scutellaris*  (Dodd & Girault, 1915) | parasitoid | Cosmopolitan | Diptera | 1981 | 5 |  |
| *Odontofroggatia galili* Wiebes, 1980 | phytophagous? | Oriental | *Ficus* | 2008 | 1 |  |
| *Ophelosia bifasciata* Girault, 1916 | parasitoid | Australasia | Hemiptera | 1966 | 6 | Berry 1995 |
| *Ophelosia charlesi* Berry, 1995 | parasitoid | Australasia | Hemiptera | 1920 | 13 | Berry 1995 |
| *Ophelosia crawfordi* Riley, 1890 | parasitoid | Australasia | Hemiptera | 1926 | 1 | Berry 1995 |
| *Ophelosia keatsi* Girault, 1927 | parasitoid | Australasia | Hemiptera | 1963 | 7 | Berry 1995 |
| *Pachyneuron aphidis* (Bouché, 1834) | parasitoid | Cosmopolitan | Hymenoptera | 1923 | x |  |
| *Pseudidarnes minerva* Girault, 1927 | phytophagous | Australasia | *Ficus* | 1993 | 1 | Early 2000; Gardner & Early 1996 |
| *Pteromalus sequester* Walker, 1835 | parasitoid | Europe | Coleoptera | 1992 |  |  |
| *Rhaphitelus maculatus* Walker, 1834 | parasitoid | Palaearctic | Coleoptera | 1966 | 2 |  |
| *Scutellista caerule* (Fonscolombe, 1832) | parasitoid | Europe | Hemiptera | 2013 | 1 | Thorpe 2013b |
| *Spalangia cameroni* Perkins, 1910 | parasitoid | Cosmopolitan | Diptera | 1962 | 3 |  |
| *Spalangia endius* Walker, 1839 | parasitoid | Cosmopolitan | Diptera | unknown | x | Valentine & Walker 1991 |
| *Spalangia ngira* Latreille, 1805 | parasitoid | Cosmopolitan | Diptera | unknown | x | Valentine & Walker 1991 |
| *Spalangia nigroaenea* Curtis, 1839 | parasitoid | Cosmopolitan | Diptera | unknown | x |  |
| *Stinoplus etearchus* (Walker, 1848) | parasitoid | Europe | Hymenoptera | 1965 | x |  |
| *Sycoscapter australis* (Froggatt, 1900) | parasitoid | Australasia | Hymenoptera | 1995 | 1 | Early 2000; Gardner & Early 1996 |
| *Theocolax formiciformis* Westwood, 1832 | parasitoid | Europe | Coleoptera | 1926 | 3 |  |
| *Trichomalopsis hemiptera* Walker, 1835 | parasitoid | Cosmopolitan | Several orders | unknown | x | Noyes 2014 |
| **Scolebythidae** |  |  |  |  |  |  |
| *Ycaploca* nr. *evansi* | parasitoid | Africa | Coleoptera | 1982 | 1 |  |
| **Scoliidae** |  |  |  |  |  |  |
| *Radumeris tasmaniensis* (Saussure, 1855) | parasitoid | Australasia | Coleoptera | 2000 | 2 | Berry et al 2001 |
| **Signiphoridae** |  |  |  |  |  |  |
| *Signiphora flavella* Girault, 1913 | parasitoid | Nearctic | Hemiptera | 1962 | 10 | Valentine & Walker 1991 |
| *Signiphora flavopalliata* Ashmead, 1880 | parasitoid | Neotropical | Hemiptera | 1977 | 1 | Love & Ferguson 1977 |
| *Signiphora merceti* Malenotti, 1916 | parasitoid | Cosmopolitan | Hemiptera | 1960 | 3 | Valentine & Walker 1991 |
| **Siricidae** |  |  |  |  |  |  |
| *Sirex noctilio* Fabricius, 1793 | phytophagous-  wood borer | Europe | Pinaceae, especially  *Pinus* | 1900 | 15 | Bain et al 2012 |
| **Sphecidae** |  |  |  |  |  |  |
| *Podalonia tydei suspiciosa* (Smith, 1856) | predator | Australasia | Lepidoptera | 1975 | 4 | Faulds 1977; Harris 1994 |
| **Tenthredinidae** |  |  |  |  |  |  |
| *Amauronematus viduatus* (Zetterstedt, 1838) | phytophagous-  leaf feeder | Holarctic | *Salix* | 2009 | 2 | Sopow 2011 |
| *Caliroa cerasi* (Linnaeus, 1758) | phytophagous-  leaf feeder | Europe | *Pyrus* and *Prunus* | 1870 | 7 | Charles 1998; Valentine 1970 |
| *Nematus oligospilus* Forster, 1854 | phytophagous-  leaf feeder | Holarctic | *Salix* | 1997 | 2 | Berry 1997b |
| *Pontania proxima* (Lepeletier, 1823) | phytophagous-  gall former | Holarctic | *Salix* | 1929 | 6 | Kay 1980 |
| *Priophorus brullei* Dahlbom, 1835 | phytophagous-  leaf feeder | Holarctic | *Rubus* spp. | 1936 | 12 | McC Callan 1978 |
| **Torymidae** |  |  |  |  |  |  |
| *Idiomacromerus terebrator* Masi, 1916 | parasitoid | Palaearctic | Hymenoptera | 1946 | 1 |  |
| *Megastigmus aculeatus* (Swederus, 1795) | phytophagous-  seed predator | Europe | *Rosa* spp | 1961 | 2 | Syrett 1990 |
| *Megastigmus* sp. | parasitoid | Australasia | *Ficus* | 1993 | 5 | Early 2000; Gardner & Early 1996 |
| *Megastigmus spermotrophus* Wachtl, 1893 | phytophagous-  seed predator | Nearctic | *Pseudotsuga* (Pinaceae; Douglas fir) | 1925 | 1 | Gourlay 1930; Bain 1977b |
| *Torymus varians* (Walker, 1833) | phytophagous-  seed predator | Palaearctic | *Crataegus* spp | 1930 | 1 | Gourley 1930; Valentine & Walker 1991 |
| **Trichogrammitidae** |  |  |  |  |  |  |
| *Trichogramma funiculatum* Carver, 1978 | parasitoid | Australasia | Lepidoptera | 1978 | 1 |  |
| *Trichogrammatoidea bactae* Nagaraja, 1978 | parasitoid | Oriental | Lepidoptera | 1975 | x |  |
| **Vespidae** |  |  |  |  |  |  |
| *Ancistrocerus gazella* (Panzer, 1798) | predator | Europe | various | 1988 | 6 | Berry 1989 |
| *Polistes chinensis antennalis* Perez, 1905 | predator | Oriental | various | 1979 | 17 | Clapperton et al 1989 |
| *Polistes humilis* (Fabricius, 1781) | predator | Australasia | various | 1896 | 8 | Valentine 1967 |
| *Vespula germanica* (Fabricius, 1793) | predator | Europe | various | 1945 | 28 | Lester et al 2013 |
| *Vespula vulgaris* (Linnaeus, 1758) | predator | Europe | various | 1978 | 28 | Lester et al 2013 |

Bain J, Sopow SL, Bulman LS (2012) The Sirex woodwasp in New Zealand: history and current status. In: The Sirex Woodwasp and its Fungal Symbiont: Research and Management of a Worldwide Invasive Pest. B. Slippers. et al. (ed), Springer Pp 167‒173.

Bain J (1977a) *Megastimus spermatrophus* Wachtl (Hymenoptera: Chalcidoidea: Torymidae). Forest and Timber insects in New Zealand. 14: 1‒4.

Bain J (1977b) *Rhicnopeltella eucalypti* Gahan (Hymenoptera: Chalcidoidea: Eulophidae), Blue-gum Chalcid. Forest and Timber insects in New Zealand. 15: 1‒4.

Withers TM (2001) Colonization of Eucalypts in New Zealand by Australian insects. Austral Ecology 26: 467‒476.

Belokobylskij SA, Austin AD (2013) New species of flightless doryctine parasitoid wasps (Hymenoptera: Braconidae: Doryctinae) from Australia and New Zealand. Australian Journal of Entomology 52: 338‒355.

Belokobylskij SA, Iqbal M, Austin A (2004) Systematics, distribution and diversity of the Australasian Doryctine wasps (Hymenoptera, Braconidae, Doryctinae). Records of the South Australian Museum Monograph series 7: 1‒150.

Berry JA (1989) *Ancistrocerus gazella* (Vespoidea: Eumenidae); a first record for New Zealand. New Zealand Entomologist 12: 63‒65.

Berry JA (1990) The New Zealand species of the subfamily Metopiinae (Hymenoptera: Ichneumonidae). New Zealand Journal of Zoology 17: 607‒614.

Berry JA (1995) Moranilini (Insecta: Hymenoptera). Fauna of New Zealand Series 33. Lincoln, Manaaki Whenua Press.

Berry JA (1997a) *Meteorus pulchricornis* (Wesmael) (Hymenoptera: Braconidae: Euphorinae), a new record for New Zealand. New Zealand Entomologist 20: 45‒48.

Berry JA (1997b) *Nematus oligospilus* (Hymenoptera: Tenthredinidae), a recently introduced sawfly defoliating willows in New Zealand. New Zealand Entomologist 20: 51‒54.

Berry JA (2007a) Alysiinae (Hymenoptera: Braconidae). Fauna of New Zealand Series 58. Lincoln, Manaaki Whenua Press.

Berry JA (2007b) Key to the New Zealand species of *Psyllaephagus* Ashmead (Hymenoptera : Encyrtidae) with descriptions of three new species and a new record of the psyllid hyperparasitoid *Coccidoctonus psyllae* Riek (Hymenoptera : Encyrtidae). Australian Journal of Entomology 46: 99‒105.

Berry JA, Osten T, Emberson RM (2001) *Radumeris tasmaniensis* (Saussure, 1855), the first record of a scoliid wasp from New Zealand. Entomofauna : Zeitschrift Für Entomologie 22: 41–48.

Berry JA, Withers TM (2002) New gall-inducing species of ormocerine pteromalid (Hymenoptera: Pteromalidae: Ormocerinae) described from New Zealand. Australian Journal of Entomology 41 (1): 18‒22.

Callan E McC (1978) Biological notes on the introduced sawfly *Priophorus morio* (Lepeptier) (Hymenoptera: Tenthredinidae) in Australia. Journal of Australian entolmological Society 17: 23‒24.

Charles JG (1998) The settlement of fruit crop arthropod pests and their natural enemies in New Zealand: an historical guide to the future. Biocontrol News and Information 19(2): 47‒58.

Clapperton BK, Moller H, Sandlant G (1989) Distribution of social wasps (Hymenoptera: Vespidae) in New Zealand in 1987. New Zealand Journal of Zoology 16: 315‒323.

Cumber RA (1959) The insect complex of sown pastures in the North Island. 5. The Hymenoptera as revealed by summer sweep-sampling. New Zealand Journal of Agricultural Research 2: 874‒897.

Don AW (2007) Ants of New Zealand. Dunedin, Otago University Press.

Donovan BJ (2007) Apoidea (Hymenoptera). Fauna of New Zealand Series 57. Lincoln, Manaaki Whenua Press.

Early JW (2000) Fig wasps (Hymenoptera: Agaonidae and Torymidae) in New Zealand. New Zealand Entomologist 23: 27‒32.

Faulds W (1977) Notes on an Australian sphecid wasp, *Podalonia suspiciosa* (Hymenoptera: Sphecidae) now established in New Zealand. New Zealand Entomologist 6(3): 312‒313.

Ferrer-Suay M, Paretas-Martínez J, Selfa J, Pujade-Villar J (2012) Charipinae fauna from New Zealand with descriptions of two new species of *Alloxysta* Förster (Hymenoptera: Cynipoidea: Figitidae: Charipinae). Australian Journal of Entomology 51: 229‒238.

Fitton MG, Gauld ID, Roberts LIN, Walker AK (1983) An African ichneumonid (Hymenoptera) in Australasia. Bulletin of Entomological Research 73: 465‒468.

Gardner RO, Early JW (1996) The naturalisation of banyan figs (*Ficus* spp., Moraceae) and their pollinating wasps (Hymenoptera: Agaonidae) in New Zealand. New Zealand Journal of Botany 34: 103‒110.

Goolsby JA, Moran P (2009) Host range of *Tetramesa romana* Walker (Hymenoptera: Eurytomidae), a potential biological control of giant reed, *Arundo donax* L. in North America. Biological Control 49: 160–168.

Gourlay ES (1930) Some parasitic Hymenoptera of economic importance in New Zealand. New Zealand Journal of Science and Technology 11: 339‒343.

Green OR (1992) New Zealand ants (Hymenoptera: Formicidae). Distribution and effects. Proceedings of the 41st annual conference of the Entomological Society of New Zealand: 67‒72.

Green OR, Gunawardana D (2006) Establishment and spread of another exotic ant *Pheidole proxima* Mayr (Hymenoptera: Formicidae) in New Zealand. The Weta 32: 16‒17.

Guerrieri E, Noyes JS (2000) Revision of European species of genus *Metaphycus* Mercet (Hymenoptera: Chalcidoidea: Encyrtidae), parasitoids of scale insects. Systematic Entomology 25: 147‒222.

Hansson C (1985) Taxonomy and biology of the Palaearctic species of *Chrysocharis* Förster, 1856 (Hymenoptera: Eulophidae). Entomologica Scandinavica (supplement) 26: 1‒130.

Harris AC (1987) Pompilidae (Insecta: Hymenoptera). Fauna of New Zealand Series 32. Lincoln, Manaaki Whenua Press.

Harris AC (1994) Sphecidae (Insecta: Hymenoptera). Fauna of New Zealand Series 12. Lincoln, Manaaki Whenua Press.

Harris RJ, Berry JA (2001) Confirmation of the establishment of three adventive ants (Hymenoptera: Formicidae) in New Zealand. New Zealand Entomologist 24: 53‒56.

Hill RL (1983) Two egg parasites (Hymenoptera: Scelionidae) of the black field cricket, *Teleogryllus commodus* (Orthoptera: Gryllidae), in New Zealand. New Zealand Journal of Zoology 10: 57‒62.

Hill RL, Wittenberg R, Gourlay AH (2001) Biology and host range of *Phytomyza vitalbae* and its establishment for the biological control of *Clematis vitalba* in New Zealand. Biocontrol Science and Technology 11: 459‒473.

Hill RL, Gordon AJ, Neser S (2000) The Potential Role of *Bruchophagus acaciae* (Cameron) (Hymenoptera: Eurytomidae) in the Integrated Control of *Acacia* Species in South Africa. Pp 919‒929. In Neal R. Spencer [ed.]. Proceedings of the X International Symposium on Biological Control of Weeds. 4‒14 July 1999, Montana State University, Bozeman, Montana, USA.

Huber JT, Fidalgo P (1997) Review of the genus *Stephanodes* (Hymenoptera: Mymaridae). Proceedings of the Entomological Society of Ontario 128: 27‒63.

Huddleston T (1986) The braconid genus *Meteorus* in New Zealand (Insecta: Hymenoptera). Journal of Natural History 20: 255‒265.

Kay MK (1980) *Pontania proxima* (Lepeletier) (Hymenoptera: Tenthredinidae), willow mining sawfly. Forest and Timber insects in New Zealand. 45: 1‒4.

Kay MK (1986) *Phylacteophaga froggatti* Riek (Hymenoptera: Pergidae), Eucalyptus leaf mining sawfly. Forest and Timber insects in New Zealand. 64: 1‒8.

Kim Il‒Kwon, McDonald M, La Salle J (2005) *Moona*, a new genus of tetrastichine gall inducers (Hymenoptera: Eulophidae) on seeds of Corymbia (Myrtaceae) in Australia. Zootaxa 989: 1–10.

Lester PJ, Beggs JB, Brown BL, Edwards ED, Groenteman R, Toft RJ, Twidle A, Ward DF (2013) The outlook for control of New Zealand’s most abundant, widespread and damaging invertebrate pests: social wasps. New Zealand Science Review 70(4): 54‒60.

Love JL, Ferguson AM (1977) Pesticide residues and greedy scale control on kiwifruit New Zealand Journal of Agricultural Research 20(1): 95‒103.

McC Callan E (1979) The Sphecidae (Hymenoptera) of New Zealand. New Zealand Entomologist 7(1): 30‒41.

Noyes JS (1988) Encyrtidae (Insecta: Hymenoptera). Fauna of New Zealand Series 13. Lincoln, Manaaki Whenua Press.

Noyes JS (2014) Universal Chalcidoidea Database. World Wide Web electronic publication. http://www.nhm.ac.uk/chalcidoids. [accessed October‒December 2014]

Olmi M (2007) New Zealand Dryinidae and Embolemidae (Hymenoptera: Chrysidoidea): new records and description of *Bocchus thorpei* new species. Records of the Auckland Museum 44: 5‒16.

Parrott AW (1954) Records of some important braconid parasites in New Zealand. New Zealand Entomologist 1: 16‒22.

Peters RS, Baur H (2011) A revision of the *Dibrachys cavus* species complex (Hymenoptera: Chalcidoidea: Pteromalidae). Zootaxa 2937: 1‒30.

Russell DA (1987) *Glabridorsum stokesii* (Cameron) (Hymenoptera: Ichneumonidae) ‒ a parasitoid of the oriental fruit moth (*Grapholita molesta*) in New Zealand. New Zealand Entomologist 10: 26‒38.

Smith F (1878) Descriptions of new species of hymenopterous insects from New Zealand, collected by Prof. Hutton, at Otago. Transactions of the Entomological Society of London 1878: 1‒7.

Soper J, Beggs JR (2013) Assessing the impact of an introduced bee, *Anthidium manicatum*, on pollinator communities in New Zealand. New Zealand Journal of Botany 51(3): 213‒228.

Sopow S (2011) Gall forming willow sawfly new to New Zealand. Forest Health News 218: 1‒2.

Swan DI (1973) Evaluation of biological control of the oak leaf‒miner *Phyllonorycter messaniella* (Zell.) (Lep., Gracillariidae) in New Zealand. Bulletin of Entomological Research 63: 49‒55.

Syrett P (1990) The rose seed chalcid *Megastigmus aculeatus* Swederus (Hymenoptera: Torymidae) on sweet brier, *Rosa rubiginosa* in the South Island tussock country. New Zealand Entomologist 13:34‒38.

Thompson WR (1955) A catalogue of the parasites and predators of insect pests. Section 2. Host parasite catalogue, Part 3. Hosts of the Hymenoptera (Calliceratid to Evaniid). pp.268 Commonwealth Agricultural Bureaux, The Commonwealth Institute of Biological Control, Ottawa, Ontario, Canada.

Thorpe S (2013a) *Cheiloneurus flaccus* (Walker, 1847) (Hymenoptera: Encyrtidae), new to New Zealand. Biodiversity Data Journal 1: e958.

Thorpe S (2013b) *Scutellista caerulea* (Fonscolombe, 1832) (Hymenoptera: Pteromalidae), new to New Zealand for the second time! Biodiversity Data Journal 1: e959.

Todd DH (1957) Incidence and parasitism of insect pests of cruciferous crops in Hawkes Bay, Wairarapa and Manawatu, 1955‒56. New Zealand Journal of Science and Technology 38: 720‒727.

Townes HK, Townes M (1981) A revision of the Serphidae (Hymenoptera). Memoirs of the American Entomological Institute 32: 1‒541.

Triapitsyn SV (2001) Review of the Australasian species of *Anagrus* (Hymenoptera Mymaridae). Belgian Journal of Entomology 3: 267‒289.

Triapitsyn SV, Berezovskiy VV (2001) Review of the Mymaridae (Hymenoptera, Chalcidoidea) of Primorskii krai: genus *Mymar* Curtis. Far Eastern Entomologist 100: 1‒20.

Valentine EW (1970) A list of the phytophagous Hymenoptera in New Zealand. The New Zealand Entomologist. 4(4): 52‒62.

Valentine EW (1967) A list of the hosts of entomophagous insects of New Zealand. New Zealand Journal of Science 10: 1100‒1209.

Valentine EW (1975) Additions and corrections to Hymenoptera hyperparasitic on aphids in New Zealand. New Zealand Entomologist 6(1): 59‒61.

Valentine EW, Walker AK (1983) Three families of Hymenoptera new to New Zealand. New Zealand Entomologist 7(4): 397‒401.

Valentine EW, Walker AK (1991) Annotated catalogue of New Zealand Hymenoptera. General Printing Services, DSIR Plant protection.

Waller JB (1982) A record of *Venturia canescens* (Hymenoptera: Ichneumonidae) in New Zealand. New Zealand Entomologist 7(3): 319.

Ward DF (2005) Changes to the classification of ants (Hymenoptera: Formicidae). The Weta 30: 16‒18.

Ward DF (2013) Revision of Bethtylidae (Hymenoptera) from New Zealand. New Zealand Entomologist 36(2): 107‒130.

Ward DF (2014) Overview and key to the New Zealand Cynipoidea (Hymenoptera). Zootaxa 3878(6): 563‒579.

Withers TM (2001) Colonization of Eucalypts in New Zealand by Australian insects. Austral Ecology 26: 467‒476.
