# Supplementary material for: Poles Apart: Comparing Trends of Alien Hymenoptera in New Zealand with Europe (DAISIE)
Source: PLoS One. 2015 Jul 6;10(7):e0132264. doi: 10.1371/journal.pone.0132264 (PMC4492945; doi:10.1371/journal.pone.0132264)
Supplement: S2 Table — “1st year” is the date a species was first recorded as established in New Zealand. “Additional sources” were used to supplement information from Ferguson et al. (2007), Gordon et al. (2010) and specimen records in the New Zealand Arthropod Collection. Last update 31/12/2014. (DOCX) [file pone.0132264.s002.docx]

**Supporting Information Table S2. List of Hymenoptera species intentionally released into New Zealand.** “1^st^ year” is the date a species was first recorded as established in New Zealand. “Additional sources” were used to supplement information from Ferguson et al. (2007), Gordon et al. (2010) and specimen records in the New Zealand Arthropod Collection. Last update 31/12/2014.

| **Family**  **Genus + Species** | **Functional**  **Group** | **Origin** | **Host** | **1st**  **Record** | **Additional Sources** |
| --- | --- | --- | --- | --- | --- |
| **Aphelinidae** |  |  |  |  |  |
| *Aphelinus mali* (Haldeman, 1851) | parasitoid | Nearctic | Hemiptera | 1921 |  |
| *Aphelinus subflavesens* (Westwood, 1837) | parasitoid | Europe | Hemiptera | 1928 |  |
| *Coccophagus gurneyi* Compere, 1929 | parasitoid | Multiple | Hemiptera | 1933 |  |
| *Coccophagus ochraceus* Howard, 1895 | parasitoid | Nearctic | Hemiptera | 1962 |  |
| *Encarsia Formosa* Gahan, 1924 | parasitoid | Neotropical | Hemiptera | 1933 |  |
| **Apidae** |  |  |  |  |  |
| *Apis mellifera* (Linneaus, 1758) | pollinator | Europe | numerous | 1839 | Donovan 2007 |
| *Bombus hortorum* (Linnaeus, 1761) | pollinator | Europe | numerous | 1906 | Donovan 2007; Gurr 1964 |
| *Bombus ruderatus* (Fabricius, 1775) | pollinator | Europe | numerous | 1906 | Donovan 2007; Gurr 1964 |
| *Bombus subterraneus* (Linneaus, 1758) | pollinator | Europe | numerous | 1885 | Donovan 2007; Gurr 1964 |
| *Bombus terrestris* (Linnaeus, 1758) | pollinator | Europe | numerous | 1885 | Donovan 2007; Gurr 1964 |
| **Braconidae** |  |  |  |  |  |
| *Alysia manducator* (Panzer, 1799) | parasitoid | Europe | Diptera | 1926 |  |
| *Apanteles subandinus* Blanchard, 1947 | parasitoid | Cosmopolitan | Lepidoptera | 1964 |  |
| *Aphidius eadyi* Stary, Gonzales & Hall, 1980 | parasitoid | Africa | Hemiptera | 1977 |  |
| *Aphidius ervi* Haliday, 1834 | parasitoid | Cosmopolitan | Hemiptera | 1977 |  |
| *Aphidius rhopalosiphi* De Stefani Perez, 1902 | parasitoid | Europe | Hemiptera | 1985 |  |
| *Ascogaster quadridentata* Wesmael, 1835 | parasitoid | Europe | Lepidoptera | 1931 |  |
| *Bracon phylacteophagus* Austin, 1989 | parasitoid | Australasia | Hymenoptera | 1988 |  |
| *Bracon variegator* Spinola, 1808 | parasitoid | Europe | Lepidoptera | 1961 |  |
| *Cotesia glomerata* (Linnaeus, 1758) | parasitoid | Europe | Lepidoptera | 1931 |  |
| *Cotesia kazak* (Telenga, 1949) | parasitoid | Europe | Lepidoptera | 1978 |  |
| *Cotesia rubercula* (Marshall, 1885) | parasitoid | Europe | Lepidoptera | 1938 |  |
| *Cotesia urabae* Austin & Allen, 1989 | parasitoid | Australasia | Lepidoptera | 2011 | Avila et al 2013 |
| *Ephedrus plagiator* (Nees von Esenbeck, 1811) | parasitoid | Oriental | Hemiptera | 1977 |  |
| *Microctonus aethiopoides* Loan, 1975 | parasitoid | Africa | Coleoptera | 1982 |  |
| *Microctonus hyperodae* Loan, 1974 | parasitoid | Neotropical | Coleoptera | 1991 | Shaw 1993 |
| *Microplitis croceipes* (Cresson, 1872) | parasitoid | Nearctic | Lepidoptera | 1987 |  |
| *Orgilus lepidus* Muesbeck, 1967 | parasitoid | Oriental | Lepidoptera | 1966 |  |
| *Pholetesor arisba* Nixon, 1973 | parasitoid | Europe | Lepidoptera | 1957 | Valentine & Walker 1991 |
| *Pholetesor circumscriptus* (Nees, 1834) | parasitoid | Europe | Lepidoptera | 1957 | Given 1959 |
| *Pholetesor pedias* (Nixon, 1973) | parasitoid | Europe | Lepidoptera | 1957 | Valentine & Walker 1991 |
| *Trioxys complanatus* Quilis, 1931 | parasitoid | Australasia | Hemiptera | 1983 |  |
| **Chalcididae** |  |  |  |  |  |
| *Brachymeria phya* (Walker, 1838) | parasitoid | Australasia | Lepidoptera | 1967 |  |
| *Brachymeria teuta* (Walker, 1841) | parasitoid | Australasia | Lepidoptera | 1967 | Noyes & Valentine 1989 |
| **Cynipidae** |  |  |  |  |  |
| *Aulacidea subterminalis* Niblett, 1946 | phytophagus-  gall former | Europe | *Hieracium* | 1999 | Klöppel et al. 2003 |
| **Encyrtidae** |  |  |  |  |  |
| *Coccidoctonus dubius* (Girault, 1915) | parasitoid | Australasia | Hemiptera | 1921 |  |
| *Copidosoma floridanum* (Ashmead, 1900) | parasitoid | Australasia | Lepidoptera | 1969 |  |
| *Habrolepis dalmanni* (Westwood, 1837) | parasitoid | Nearctic | Hemiptera | 1923 |  |
| *Metaphycus lounsburyi* (Howard, 1898) | parasitoid | Australasia | Hemiptera | 1922 |  |
| *Microterys nietneri* (Motschulsky, 1859) | parasitoid | Australasia | Hemiptera | 1921 |  |
| *Pseudaphycus maculipennis* Mercet, 1923 | parasitoid | Australasia | Hemiptera | 2001 |  |
| *Tachinaephagus zealandicus* Ashmead, 1904 | parasitoid | Australasia | Diptera | 1926 |  |
| *Tetracnemoidea brevicornis* (Girault, 1915) | parasitoid | Nearctic | Hemiptera | 1920 | Dumbleton 1936;  Valentine & Walker 1991 |
| **Eulophidae** |  |  |  |  |  |
| *Achrysocharoides latreillii* (Curtis, 1826) | parasitoid | Europe | Lepidoptera | 1957 | Given 1959 |
| *Pediobius epigonus* (Walker, 1839) | parasitoid | Europe | Diptera | 1894 |  |
| *Pediobius metallicus* (Nees, 1834) | parasitoid | Europe | Diptera | 1894 |  |
| *Thripobius javae* (Girault, 1917) | parasitoid | Nearctic | Thysanoptera | 2001 |  |
| **Halictidae** |  |  |  |  |  |
| *Nomia melanderi* Cockerell, 1906 | pollinator | Nearctic | Lucerne | 1964 | Donovan 2007 |
| **Ibaliidae** |  |  |  |  |  |
| *Ibalia leucospoides* (Hockenwarth, 1785) | parasitoid | Europe | Hymenoptera | 1954 |  |
| **Ichneumonidae** |  |  |  |  |  |
| *Diadegma semiclausum* (Hellen, 1949) | parasitoid | Europe | Lepidoptera | 1936 |  |
| *Diadromus collaris* (Gravenhorst, 1829) | parasitoid | Europe | Lepidoptera | 1938 |  |
| *Glabridorsum stokesii* (Cameron, 1912) | parasitoid | Australasia | Lepidoptera | 1967 |  |
| *Lathrolestes luteolator* (Gravenhorst, 1829) | parasitoid | Europe | Hymenoptera | 1923 |  |
| *Liotryphon caudatus* (Ratzeburg, 1848) | parasitoid | Multiple | Lepidoptera | 1906 |  |
| *Mastrus ridens* (Horstmann, 2009) | parasitoid | Europe | Lepidoptera | 2012 |  |
| *Megarhyssa nortoni* (Cresson, 1864) | parasitoid | Nearctic | Hymenoptera | 1962 |  |
| *Rhyssa persuasoria* (Linnaeus, 1758) | parasitoid | Europe | Hymenoptera | 1929 |  |
| *Sphecophaga vesparum vesparum* (Curtis, 1828) | parasitoid | Europe | Hymenoptera | 1985 |  |
| *Temelucha* sp. | parasitoid | Oriental | Lepidoptera | 1966 |  |
| *Xanthopimpla rhopaloceros* Krieger, 1914 | parasitoid | Australasia | Lepidoptera | 1967 |  |
| **Megachilidae** |  |  |  |  |  |
| *Megachile rotundata* (Fabricius, 1787) | pollinator | Nearctic | Alfalfa (lucerne) | 1971 | Donovan 2007 |
| *Osmia coerulescens* (Linnaeus, 1758) | pollinator | Europe | Red clover | 1996 | Donovan 2007 |
| **Mymaridae** |  |  |  |  |  |
| *Anaphes nitens* (Girault, 1928) | parasitoid | Australasia | Coleoptera | 1927 |  |
| **Platygastridae** |  |  |  |  |  |
| *Inostemma boscii* (Jurine, 1807) | parasitoid | Europe | Diptera | 1938 | Dumbleton 1964 |
| *Platygaster demades* Walker, 1835 | parasitoid | Europe | Diptera | 1925 |  |
| *Trissolcus basalis* (Wollaston, 1858) | parasitoid | Cosmopolitan | Hemiptera | 1949 |  |
| **Pteromalidae** |  |  |  |  |  |
| *Enoggera nassaui* Girault, 1915 | parasitoid | Australasia | Coleoptera | 1987 |  |
| *Muscidifurax raptor* Girault & Sanders, 1910 | parasitoid | Nearctic | Diptera | 1969 |  |
| *Nasonia vitripennis* (Walker, 1836) | parasitoid | Australasia | Diptera | 1922 |  |
| *Pteromalus puparum* (Linnaeus, 1758) | parasitoid | Europe | Lepidoptera | 1932 |  |
| *Pteromalus semotus* (Walker, 1834) | parasitoid | Europe | Lepidoptera | 1953 |  |

Avila GA, Berndt LA, Holwell GI (2013) First releases and monitoring of the biological control agent, *Cotesia urabae* Austin and Allen (Hymenoptera: Braconidae). New Zealand Entomologist 36(2): 65‒72.

Donovan BJ (2007) Apoidea (Hymenoptera). Fauna of New Zealand Series 57. Lincoln, Manaaki Whenua Press.

Dumbleton LJ (1936) The biological control of fruit pests in New Zealand. New Zealand Journal of Science and Technology. 18: 588‒592.

Dumbleton LJ (1964) Notes on insects. New Zealand Entomologist 3(3): 24‒25.

Ferguson CM, Moeed A, Barratt BIP, Hill RL, Kean JM (2007) BCANZ - Biological Control Agents introduced to New Zealand. http://www.b3nz.org/bcanz (accessed January 2015).

Given BB (1959) Biological control factors influencing populations of oak leaf-miner, *Lithocolletis messaniella* Zeller, in New Zealand including the introduction of parasites. New Zealand Journal of Agricultural Research 2: 124‒133.

Gordon DP (2010) New Zealand Inventory of Biodiversity. Volume 2. Kingdom Animalia. Chaetognatha, Ecdysozoa, Ichnofossils. Canterbury University Press, Christchurch.

Gurr L (1964) The distribution of bumblebees in the South Island of New Zealand. New Zealand Journal of Science 7: 625-642.

Klöppel M, Smith L, Syrett P (2003) Predicting the impact of the biocontrol agent *Aulacidea subterminalis* (Cynipidae) on growth of *Hieracium pilosella* (Asteraceae) under differing environmental conditions in New Zealand. Biocontrol Science and Technology 13: 207‒218.

Noyes JS Valentine EW (1989) Chalcidoidea (Insecta: Hymenoptera) introduction, and review of genera in smaller families. Fauna of New Zealand Series 18. Lincoln, Manaaki Whenua Press.

Shaw SR (1993) Three new *Microctonus* species indigenous to New Zealand (Hymenoptera: Braconidae). New Zealand Entomologist 16: 29‒39.

Valentine EW, Walker AK (1991) Annotated catalogue of New Zealand Hymenoptera. General Printing Services, DSIR Plant protection.
